# The Application of an Extracellular Vesicle-Based Biosensor in Early Diagnosis and Prediction of Chemoresponsiveness in Ovarian Cancer

**DOI:** 10.3390/cancers15092566

**Published:** 2023-04-30

**Authors:** Meshach Asare-Werehene, Robert A. Hunter, Emma Gerber, Arkadiy Reunov, Isaiah Brine, Chia-Yu Chang, Chia-Ching Chang, Dar-Bin Shieh, Dylan Burger, Hanan Anis, Benjamin K. Tsang

**Affiliations:** 1 Departments of Obstetrics & Gynecology and Cellular & Molecular Medicine, Centre for Infection, Immunity and Inflammation, Interdisciplinary School of Health Sciences, University of Ottawa, Ottawa, ON K1N 6N5, Canada; 2 Chronic Disease Program, Ottawa Hospital Research Institute, The Ottawa Hospital, Ottawa, ON K1Y 4E9, Canada; 3 School of Electrical Engineering and Computer Science, University of Ottawa, Ottawa, ON K1N 6N5, Canada; 4 Ottawa-Carleton Institute for Biomedical Engineering, University of Ottawa, Ottawa, ON K1N 6N5, Canada; 5 Department of Biology, St. Francis Xavier University, 2320 Notre Dame Avenue, Antigonish, NS B2G 2W5, Canada; 6 Department of Biological Science and Technology, National Yang Ming Chiao Tung University, Hsinchu 30068, Taiwan; 7 Center for Intelligent Drug Systems and Smart Bio-Devices (IDS2B), National Yang Ming Chiao Tung University, Hsinchu 30068, Taiwan; 8 Department of Electrophysics, National Yang Ming Chiao Tung University, Hsinchu 30010, Taiwan; 9 Institute of Physics, Academia Sinica, Taipei 10529, Taiwan; 10 Institute of Basic Medical Science, Institute of Oral Medicine and Department of Stomatology, National Cheng Kung University Hospital, National Cheng Kung University, Tainan 704, Taiwan; 11 Advanced Optoelectronic Technology Center and Center for Micro/Nano Science and Technology, National Cheng Kung University, Tainan 701, Taiwan

**Keywords:** ovarian cancer (OVCA), plasma gelsolin (pGSN), chemoresistance, extracellular vesicles (EVs), cisplatin (CDDP), cortactin (CTTN), CA125

## Abstract

**Simple Summary:**

Late diagnosis and chemoresistance are key obstacles to ovarian cancer treatment success. Thus, there exists a need to develop new markers to detect ovarian cancer at an early stage as well as predict chemoresistance. We have developed a nanosensor platform that reacts with extracellular vesicles and cisplatin as well as predicts early-stage cancers and chemoresistance. Mechanistically, we have shown that chemoresistant OVCA cells produce large amounts of plasma gelsolin (pGSN) that induces increased production of small extracellular vesicles as a means of exporting cisplatin from the cell. This helps to prevent cisplatin-induced apoptosis in chemoresistant cells.

**Abstract:**

Background: Ovarian cancer (OVCA) is the most fatal gynecological cancer with late diagnosis and plasma gelsolin (pGSN)-mediated chemoresistance representing the main obstacles to treatment success. Since there is no reliable approach to diagnosing patients at an early stage as well as predicting chemoresponsiveness, there is an urgent need to develop a diagnostic platform for such purposes. Small extracellular vesicles (sEVs) are attractive biomarkers given their potential accuracy for targeting tumor sites. Methods: We have developed a novel biosensor which utilizes cysteine-functionalized gold nanoparticles that simultaneously bind to cisplatin (CDDP) and plasma/cell-derived EVs, affording us the advantage of predicting OVCA chemoresponsiveness, and early diagnosis using surface-enhanced Raman spectroscopy. Results: We found that pGSN regulates cortactin (CTTN) content resulting in the formation of nuclear- and cytoplasmic-dense granules facilitating the secretion of sEVs carrying CDDP; a strategy used by resistant cells to survive CDDP action. The clinical utility of the biosensor was tested and subsequently revealed that the sEV/CA125 ratio outperformed CA125 and sEV individually in predicting early stage, chemoresistance, residual disease, tumor recurrence, and patient survival. Conclusion: These findings highlight pGSN as a potential therapeutic target and provide a potential diagnostic platform to detect OVCA earlier and predict chemoresistance; an intervention that will positively impact patient-survival outcomes.

## 1. Introduction

Ovarian cancer (OVCA) remains the most fatal gynecological cancer with a 5-year survival rate of 45% primarily due to late diagnosis and chemoresistance [1,2,3]. Although patients initially respond to treatment (primary surgical debulking and chemotherapy), about 50–70% of patients relapse and become resistant to further treatment [3]. The 5-year survival rate for stage 1 patients is >90%, whereas the 5-year survival rates for stage 2, 3 and 4 patients are ~70%, ~39%, and ~17%, respectively [1,3]. However, only about 19% of ovarian cancer patients are diagnosed at the early stage despite the use of conventional biomarkers such as CA125, HE4, ROMA, and OVA1 [1,2,3,4,5,6]. This suggests an urgent need for highly reliable and effective diagnostic platforms for early-stage disease and chemoresistant prediction.

Plasma gelsolin (pGSN; also known as secreted GSN) is a multi-functional actin-binding protein and the secreted isoform of the GSN gene [7,8,9]. pGSN is implicated in the progression, chemoresistance, and metastasis of a plethora of cancer types including OVCA [10,11,12,13,14,15,16,17,18]. We have previously demonstrated that pGSN is highly expressed in chemoresistant OVCA tumors and transported via small extracellular vesicles (sEVs) [10,15,16]. sEV-pGSN autoregulates its own gene expression and confers resistance to chemosensitive cells in a paracrine manner through the activation of the FAK/AKT/HIF1a pathway [10]. Additionally, sEV-pGSN induces caspase-3-dependent apoptosis and suppresses the anti-tumor functions of immune cells such as CD8+ T cells, CD4+ T cells, and M1 macrophages [15,16]. Although pGSN is closely associated with sEV secretion, we have yet to determine the role of pGSN in sEV secretion.

EVs present as an attractive biomarker given that they carry bioprints of the secreting cells and can capture the molecular landscape of cancer cells. This offers a superior advantage for cancer diagnostic tests compared with other biomarkers. EVs are secreted from most cells and are also present in liquid biopsies such as urine, plasma, and ascites. EVs are broadly categorized based on their origin, size, release pathway, biogenesis and function. Based on their mode of biogenesis, EVs can be sub-classified as ectosomes (cellular microparticles or microvesicles), exosomes and apoptotic EVs and serve as carriers for transporting molecular signatures such as proteins, RNA, DNA, and miRNAs [19,20]. The contents of the cargo can induce phenotypic and genetic changes in the recipient cells. sEVs play a key role in tumorigenesis and chemoresistance in different cancer types [15,21,22,23].

Chemoresistant OVCA cells secrete increased levels of sEV-CDDP and sEVs containing pGSN, conferring chemoresistance on otherwise chemosensitive cells as well as downregulating the tumor-killing functions of immune cells in the tumor microenvironment [10,15,16]. Despite the potential diagnostic utility of sEVs, detecting EV-specific antigens using conventional techniques such as polymerase chain reaction (PCR) and enzyme-linked immunosorbent assay (ELISA) present as a major obstacle in cancer diagnostics. Thus, the use of technologies that could effectively detect the biochemical fingerprints of EVs would significantly enhance their clinical utility as opposed to targeting specific markers.

Surface-enhanced Raman spectroscopy (SERS) offers the capacity to examine the biochemical structure of biological analytes and presents as a useful tool in differentiating cancer cells from normal cells [24,25]. Interestingly, the application of SERS in liquid biopsy analyses has encountered major setbacks due to the heterogeneity of samples and interferences from large proteins. Similar obstacles are likely to be encountered in chemoresistance prediction given that not all circulating EVs originate from cancer cells, and some share biochemical characteristics with the target EVs. We have recently developed a novel sensor which utilizes cysteine-functionalized gold nanoparticles to simultaneously bind to cisplatin (CDDP) and sonicated sEVs affording us the advantage of quantifying EVs and EV-CDDP with high accuracy using SERS [26]. This unique biosensor will enable us to overcome the challenges of tumor heterogeneity and protein interferences.

In this study, we investigated the application of an EV-based novel biosensor in the diagnosis of early-stage OVCA and the prediction of chemoresistance. Additionally, we examined the regulation of EV-mediated CDDP efflux in chemoresistant OVCA cells by pGSN, given the positive association between pGSN and sEVs. We found that pGSN is a key regulator of EV secretion and EV-mediated release of CDDP from chemoresistant OVCA cells. Furthermore, sEV/CA125 is a strong indicator for stage 1 OVCA and a predictor of chemoresistance.

## 2. Materials and Methods

### 2.1. Plasma Samples

A total of 99 ovarian cancer patients (high-grade serous, HGS; 69, low-grade serous, LGS; 4, not verified; 26) with predetermined CA125 levels and 20 healthy non-cancerous subjects provided plasma samples for extracellular vesicle (EV) isolation, characterization, and analyses. Patients were recruited at the CHUM from 1992–2012 and did not receive any neoadjuvant chemotherapy or radiotherapy. All patients were managed with primary surgery. Gynecologic-oncologic pathologists examined all samples and assigned tumor grade and histologic subtypes in accordance with the International Federation of Gynecology and Obstetrics (FIGO) criteria. Computed Tomography imaging and CA125 levels during follow-ups were used to define disease-free survival (DFS; time of diagnosis to time of recurrence) and overall survival (OS; time of diagnosis to time of death). Details of patient demographics and clinical outcomes are outlined in Supplementary Materials Table S1.

### 2.2. Interrogation of OVCA Public Datasets

Ovarian cancer public datasets were interrogated using http://www.rocplot.org/ovarian/index (accessed on 16 June 2022 ) and http://gepia.cancer-pku.cn/index.html on (accessed on 12 December 2022). The differential expressions (box plot; chemoresistance vs. chemosensitive) and test performances (ROC curves; chemoresistance prediction) of GSN, CTTN, ABCB1, MRP2, RAB27A, BLOC1S1, BLOC1S2, BLOC1S3, BLOC1S4, BLOC1S5, BLOC1S6, DTNBP1, RAB32, RAB38, SNAPIN, VPS11, VPS16, VPS18, VPS33A, VPS33B, VPS39 and VPS41 (*n* = 958; sensitive = 862; resistant = 96; platinum) were investigated. Spearman’s correlation tests were performed to assess the association between pGSN and the other genes. Significant correlations were inferred as *p* ≤ 0.05 (Table S2)

### 2.3. Reagents

Cis-diaminedichloroplatinum (CDDP), phenylmethylsulfonyl fluoride (PMSF), aprotinin, dimethyl sulfoxide (DMSO), sodium orthovanadate (Na_3_VO_4_), CCK-8, and Hoechst 33,258 were supplied by Millipore Sigma (St. Louis, MO, USA). Two preparations of pGSN siRNA (siRNA1 and 2) and scrambled sequence siRNA (control) were purchased from Integrated DNA Technology (Coralville, IA, USA) and Dharmacon (Lafayette, CO, USA), respectively. Human recombinant plasma gelsolin (hrpGSN) was synthesized and provided by Dr. Chia-Ching Chang, National Yang Ming Chiao Tung University, Taiwan. pGSN cDNA and 3.1A vector plasmids were provided by Dr. Dar-Bin Shieh, National Cheng Kung University Hospital, Taiwan. See Table S3 for details on antibodies and other reagents.

### 2.4. Ovarian Cancer Cell Lines

Chemosensitive and chemoresistant OVCA cell lines (1.6 × 10^6^ cells) of high-grade serous (HGS; TOV3041G, TOV3133 and OV90) and endometrioid (A2780s and A2780cp) histologic subtypes were used for all in vitro studies. Dr. Anne-Marie Mes-Masson (CHUM, Montreal, QC, Canada) generously donated the HGS cell lines and the endometrioid cell lines were generously donated by Dr. Barbara Vanderhyden (Ottawa Hospital Research Institute, Ottawa, ON, Canada). Quality control was performed to prevent any batch-to-batch changes on the morphology and growth rate. In addition, cell lines were regularly authenticated and tested for *Mycoplasma* contamination using PlasmoTest^TM^ Mycoplasma Detection kit (InvivoGen, San Diego, CA, USA; catalog number: rep-pt1). The HGS OVCA cells were maintained in OSE medium (wisent Inc., St-Bruno, QC, Canada) supplemented with 10% FBS (Millipore Sigma; St. Louis, MO, USA), 250 μg/mL amphotericin B, and 50 mg/mL gentamicin (wisent Inc., St-Bruno, QC, Canada). The endometrioid OVCA cells were maintained in Gibco RPM1 1640 (Life Technologies, Grand Island, NY, USA) supplemented with 10% FBS (Millipore Sigma; St. Louis, MO, USA), 50 U/mL penicillin, 50 U/mL streptomycin, and 2 mmol/L l-glutamine (Gibco Life Technologies, NY, USA). All experiments were carried out in serum-free media. Details on the mutations of the cell lines used are described in Supplementary Materials Table S4.

### 2.5. pGSN Gene Interference

Chemoresistant cells were transfected with siRNA (50 nM, 24 h; scramble as controls) and sensitive cells transfected with cDNA (2 µg; 24 h; empty vectors as controls) using lipofectamine 2000, and were subsequently treated with CDDP (10 µM; 24 h)) then harvested for analysis as previously described [27,28,29]. Two different siRNAs were used for pGSN target to exclude off-target effects. Western blot was used to confirm pGSN knock-down and over-expression [30]. See Table S3 for details on antibodies and Table S5 for customized siRNA oligonucleotide duplexes.

### 2.6. Extracellular Vesicle (EVs) Isolation and Characterization

EVs were isolated and characterized using serum-free conditioned media from cultured cells as well as ovarian cancer patient plasma as described previously [31]. Differential ultracentrifugation was used to isolate EVs from conditioned media while ExoQuick Ultra was used for the plasma samples. Cancer-cell-derived conditioned media were centrifuged at 300× *g* (10 min at RT) to remove cells and debris, 20,000× *g* (20 min at RT) to remove large EVs (microparticles), and then at 100,000× *g* (90 min at 4 °C) to pellet sEVs. For plasma-derived EV isolation, 100 μL of ExoQuick precipitation reagent is added to plasma (40 μL in 500 μL of 0.1 μm filtered PBS) and incubated on ice for 30 min. The content is then centrifuged at 3000× *g* for 10 min and the suspension discarded. The pellet is then re-suspended in a buffer (provided by the manufacturer), transferred into pre-washed ExoQuick Ultra columns, and then centrifuged at 1000× *g* for 30 secs. The receiving tube is then detached and EVs collected. EVs are then characterized using nanoparticle tracking analyses (particle size distribution and concentration), Western blot (EV-specific markers) and transmission electron microscopy (EV size and purity). Isolated EVs that were not used immediately were suspended in PBS and stored at −80 °C for subsequent analyses.

### 2.7. Nanoparticle Tracking Analysis (NTA)

EVs diluted in PBS were analyzed, using the ZetaView PMX110 Multiple Parameter Particle Tracking Analyzer (Particle Metrix, Meerbusch, Germany) in size mode, and ZetaView software version 8.02.28, as previously described [31,32]. With 11 camera positions, EVs were captured at 21 °C.

### 2.8. Protein Extraction and Western Blot Analysis

Western blotting (WB) procedure for proteins was carried out as described previously [27,28,30]. After protein transfer, membranes were incubated with primary antibodies (1:1000) in 5% (*wt*/*vol*) blotto and subsequently with the appropriate horseradish peroxidase (HRP)-conjugated secondary antibody (1:2000) in 5% (*wt*/*vol*) blotto. See Supplementary Materials Table S3 for details of antibodies used. Chemiluminescent Kit (Amersham Biosciences, Amersham, UK) was used to develop protein bands and visualized by BioRad ChemiDoc MP. Signal intensities generated were measured densitometrically using Image J. Original, uncropped Western blot membrane can be found in Figure S6. 

### 2.9. Assessment of Cell Proliferation and Apoptosis

Apoptosis and cell proliferation were assessed morphologically with Hoechst 33,258 nuclear stain and colorimetrically with the CCK-8 assay, respectively. “Blinded” counting approach was used to prevent experimental bias with the Hoechst 33,258 nuclear staining.

### 2.10. Transmission Electron Microscopy (TEM)

OVCA cells were pelleted (4000 g; 20 min) and processed, as previously described [33]. Resin sections were stained with uranyl acetate and lead citrate solutions and examined with a Jeol JEM 1230 transmission electron microscope (Akishima, Japan).

### 2.11. Immunoelectron Microscopy (iEM)

Cell pellets (4000 g; 20 min) were processed as previously described [33]. The grids were washed three times in PBST, immunostained with anti-pGSN antibody (Supplementary Materials Table S3), rinsed in distilled water, stained with uranyl acetate and lead citrate, and photographed with a Jeol JEM 1230 transmission electron microscope (Akishima, Japan).

### 2.12. EDS and ICP-MS

Energy Dispersive Spectroscopy (EDS) was performed in High-Resolution Transmission Electron Microscopy (HR-TEM, JEM-2010/JEOL Co., 200 KV) in NCKU for the quantitative analysis of elemental compositions and distributions in chemoresistant cells, particularly platinum signal representing the anti-cancer drug. The samples were dispersed in ethanol and dropped onto a copper grid with an amorphous carbon film followed by evaporation of the solvent in a vacuum desiccator. The elemental composition was detected and quantified by EDS. To determine the cisplatin content in the sEVs and in the condition medium, the Pt ion present in the drug was analyzed by *THERMO Element XR* High-resolution ICP-mass spectrometer (*Element XR*, Thermo Fisher Scientific, Bremen, Germany). Conditioned media were collected and sEVs were isolated using differential ultra-centrifugation before analysis. The sEVs pellets were lysed with lysis buffer (1% Triton X-100, 0.1 M Tris-HCl pH 7.4, 0.1% SDS) containing protease inhibitors (Sigma-Aldrich). Then, the sEVs lysates were digested by adding 200 µL of concentrated nitric acid (Optima Grade, Fisher Scientific, Cambridge, MA, USA) and kept at 60 °C for 2 h. The final digested solutions were diluted with deionized water (DI Water). Indium (1 µg/L) was added to the specimens as internal standard. The same matrix (lysing solution, nitric acid) was used as the calibration standard for the external control. Cell culture medium samples were diluted 1:100 with DI Water before performing the Pt analysis, adding only Indium as internal standard to minimize the effect of instrumental variation. The levels of the cisplatin into the medium were expressed in ng per mL.

### 2.13. SERS Quantification of sEV and CDDP

Cysteine-capped gold nanoparticles were synthesized using the citrate reduction method to form particles from Au^3+^. After the particles had formed, they were washed via centrifugation and re-suspended in 10 mM NaOH with 1 µM cysteine. The amino acid preferentially bound to the gold surfaces due to the formation of a gold-thiol bond. To analyze sEV samples, they were first sonicated for 15 min to break-up the vesicles before mixing with the nanoparticles and incubating overnight. Before Raman measurement, NaCl was added to the sEV-nanoparticle mixture to initiate aggregation, and this process was measured by means of an in-house built Raman spectrometer (785 nm excitation, 30 mW, 0.65 NA objective, Kaiser f/18i spectrograph, TE cooled Andor CCD). CDDP concentration could be inferred based on the aggregation rate of the particles, with increasing drug concentration resulting in faster aggregation [26]. The steady-state SERS spectrum contained the signature of the sEV components which had also bound to the nanoparticles, and this spectrum could be used to quantify the sEVs in the sample using multi-variate regression [26].

### 2.14. Statistical Analyses

The SPSS software version 25 (SPSS Inc., Chicago, IL, USA) and Graphpad Prism 7 (San Diego, CA, USA) were used to perform all statistical analyses and two-sided *p* ≤ 0.05 considered to indicate statistical significance. Receiving operating characteristic (ROC) curves were used to assess the performances of sEV/CA125, sEV and CA125 over their entire range of values. The area under the curve (AUC) was used as an index of global test performance. The association between sEVs and CA125 was analysed using Pearson’s correlation test (two-tailed). The means of sEVs and sEV/CA125 levels were plotted against stage, residual disease, histological subtypes, tumor recurrence, chemoresistance and survival using scatter plots and statistical analyses performed via unpaired *t*-test; Gaussian distribution was tested. The relationship of these dichotomous variables to other clinicopathologic correlates was examined using Fisher exact test, *t*-test and Kruskal–Wallis Test as appropriate. Survival curves (DFS and OS) were plotted with Kaplan–Meier and *p*-values calculated using the log-rank test.

## 3. Results

### 3.1. Extracellular Vesicle Isolation and Characterizations from OVCA Cell Lines

**Figure 1:**
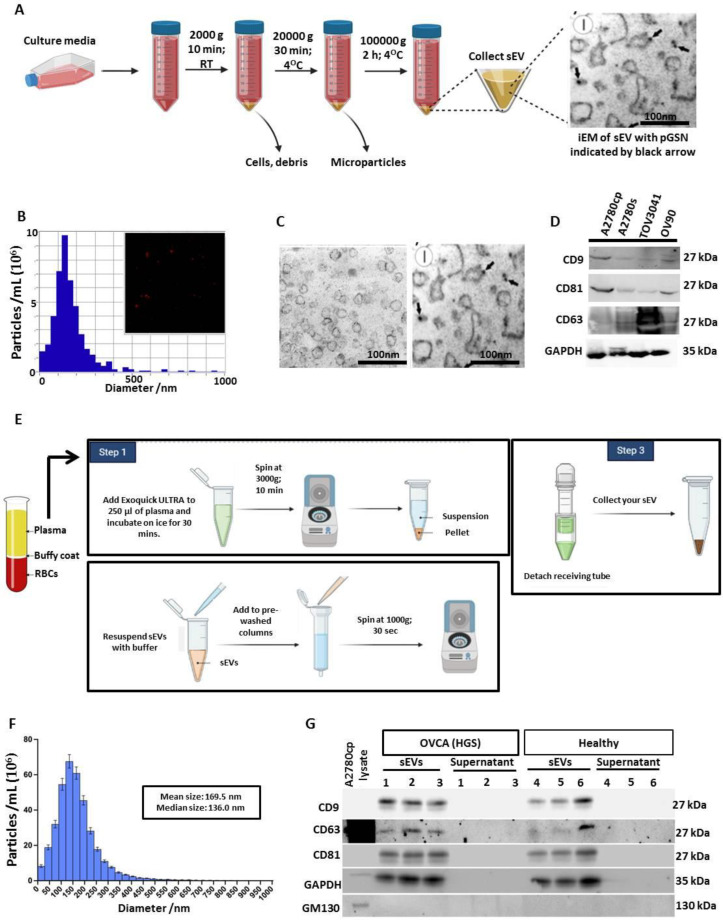
Extracellular vesicle (EVs) isolation and characterization. (**A**) Conditioned media form cultured OVCA cells were collected and filtered using 0.22 μM filter. Differential ultra-centrifugation was used to isolate small EVs after which they were characterized using (**B**) nanoparticle-tracking analyses (NTA; particle size distribution and concentration), (**C**) electron microscopy (EM) and (**D**) Western blot (CD9, CD81, CD63, GAPDH). (**E**) Small EVs were isolated from the plasma of human OVCA patients (*n* = 99) and non-OVCA subjects (*n* = 20) using Exoquick Ultra technique. EVs were then characterized using (**F**) nanoparticle-tracking device (size distribution) and (**G**) Western blot analyses (small EV markers; CD9, CD63, CD81, negative marker; GM130, vesicle integrity marker; GAPDH).

To investigate the role of pGSN in EV-mediated secretion of CDDP in chemoresistant OVCA, OVCA cell lines of HGS and endometrioid subtypes were cultured with and without CDDP treatment (10 μM; 24 h) and their conditioned media were collected for EV isolation and characterization. Conditioned media were filtered with 0.22 μM pore size filter and EVs isolated using differential ultra-centrifugation (Figure 1A). EVs were characterized using nanoparticle-tracking analyses to determine particle size distribution and concentration (Figure 1B), immune-gold electron microscopy to determine particle size, EV purity and gelsolin content (Figure 1C). Based on the particle isolation and characterizations, the EVs had an average size of 130 nm and were positive for CD63, CD9 and CD81 markers suggesting that they are sEVs (Figure 1D). The presence of GAPDH also suggests the sEVs were intact (Figure 1D). SEVs from the plasma of OVCA patients and non-OVCA subjects were isolated using ExoQuick ULTRA (System Biosciences), which employs the use of a polymer for robust vesicle precipitation, low-speed centrifugation, and column filtration to minimize protein aggregates (Figure 1E). SEVs were characterized using nanoparticle-tracking analysis and Western blotting (Figure 1F,G). Unlike the sEV-free plasma, the sEVs isolated from patients’ plasma had an average median size of 136 nm and were positive for sEV markers (CD9, CD63, and CD81) but negative for GM130, suggesting that they are mainly sEVs (Figure 1F,G). The presence of GAPDH also suggests the sEVs were intact (Figure 1G).

### 3.2. Chemoresistant OVCA Cells Express High pGSN and CTTN and Are Associated with Increased sEV-CDDP

**Figure 2:**
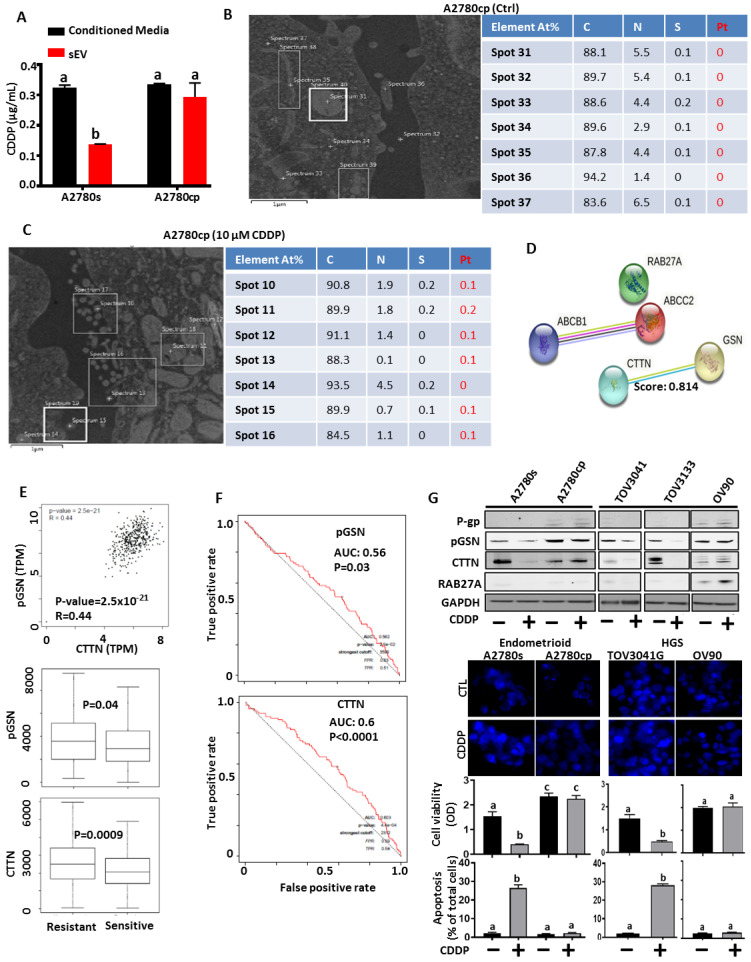
Chemoresistant OVCA cells express high pGSN and CTTN and are associated with increased sEV-CDDP. (**A**) sEV-CDDP content is higher in chemoresistant cells compared with chemosensitive cells. Chemosensitive (A2780s) and chemoresistant (A2780cp) cells were cultured and treated with or without CDDP (10 μM; 24 h). Conditioned media were collected and sEVs isolated using differential ultra-centrifugation. CDDP content in the sEVs and conditioned media was analyzed using inductively coupled plasma reactive ion etching (ICP). (**B**,**C**) Chemoresistant OVCA cells were treated with or without CDDP (10 μM; 24 h). Platinum (Pt), calcium (C), nitrogen (N) and sulphur (S) distribution within chemoresistant cells were analyzed using energy dispersive X-ray spectroscopy (EDS). (**D**) Using string-db.org, we found a strong interaction between pGSN and CTTN but not ABCB1 (p-gp), ABCC2, RAB27A. (**E**,**F**) Our investigation of TCGA public dataset revealed that pGSN positively correlates with CTTN expression and both proteins are highly elevated in chemoresistant OVCA patients (*n* = 958). The test performances of pGSN and CTTN were evaluated using ROC curves and significant predictions were observed. (**G**) Chemosensitive (A2780s, TOV3041G, TOV3133) and chemoresistant (A2780cp and OV90) cells were treated with or without CDDP (10 μM; 24 h). pGSN, CTTN, P-gp, RAB27A and GAPDH contents were assessed by Western blot. Cisplatin-induced cell death was analyzed by Hoechst staining and CCK-8 assay. Results are expressed as means ± SD from three independent replicate experiments. [(**A**), a; * *p* < 0.05 vs. b; (**G**), (a; ** *p* < 0.01 vs. b, b; *** *p* < 0.001 vs. c)].

Chemosensitive (A2780s) and chemoresistant (A2780cp) cells were treated with or without CDDP (10 μM; 24 h) in serum-free conditioned media (Figure 2A–C). The conditioned media were collected and sEVs isolated. CDDP concentrations were determined in the sEVs as well as EV-free conditioned media using inductively coupled plasma reactive ion etching (ICP) (Figure 2A). We observed that sEVs derived from chemoresistant cells contained significantly higher levels of CDDP compared with that of chemosensitive cells (Figure 2A). Meanwhile, there were no significant differences in the CDDP concentration detected in the serum-free conditioned media of both cells (Figure 2A). We also assessed the platinum distribution in the chemoresistant cells after CDDP treatment using energy dispersive X-ray spectroscopy (EDS) (Figure 2B,C). Cellular distribution of platinum was not significantly different between chemoresistant cells treated with CDDP and those not treated (Figure 2B,C), suggesting that CDDP might have been exported via sEV secretion.

SEV-mediated secretion of CDDP is a key determinant of OVCA chemoresistance; however, the underlying mechanism is not known. Given that pGSN has been shown to be a marker of chemoresistance in OVCA as well as play a major role in EV biology; we investigated its potential role in sEV-mediated OVCA chemoresistance. We interrogated string-db.org and found that while there was no interaction between pGSN and drug transport proteins (p-glycoprotein; ABCB1 and ABCC2) as well as RAB27A, there was a potential interaction between pGSN and CTTN (Figure 2D). We further interrogated the ovarian cancer TCGA public dataset (http://gepia.cancer-pku.cn/index.html, accessed on 16 June 2022) to assess the correlation (Spearman coefficient) between pGSN and CTTN, RAB27A, or ABCB1. We found that pGSN positively correlated with CTTN and both proteins were significantly elevated in chemoresistant tumors compared with chemosensitive tumors (Figure 2E and Figure S1). A positive correlation was found between pGSN and MRP2, p-gp, and RAB27A (Figure S1B,C). Additionally, we interrogated OV datasets (http://www.rocplot.org/ovarian/index, accessed on 16 June 2022) to determine the differential expression (box plot; chemoresistance vs. chemosensitive) and test performance (receiver operating characteristic (ROC) analysis; chemoresistance prediction) of GSN, CTTN, ABCB1, MRP2, and RAB27A (Figure 2E and Figure S1) (*n* = 958; sensitive = 862; resistant = 96). All patients had serous tumors, had received platin-based treatment, and response was based on progression-free survival at 6 months. Using ROC analysis, we examined the test performance of pGSN (area under the curve (AUC) = 0.56; *p* = 0.03) and CTTN (AUC: 0.6; *p* < 0.0001) and found both markers are significantly predictive of OVCA chemoresistance (Figure 2F). We observed that P-glycoprotein, but neither MRP2 nor RAB27A, were significantly predictive of chemoresistance in human OVCA tumors (Supplementary Materials Figure S1A).

To begin investigating the role of sEV-mediated chemoresistance in OVCA, chemosensitive (A2780s, TOV3041G, TOV3133) and chemoresistant (A2780cp and OV90) OVCA cells were treated with or without CDDP (10 μM; 24 h) (Figure 2G). pGSN, CTTN, P-gp, RAB27A and GAPDH contents were assessed by Western blot. Cisplatin-induced cell death was analyzed by Hoechst staining, and cell viability was verified by CCK-8 assay (Figure 2G). pGSN and CTTN contents were reduced by CDDP in the chemosensitive cells but not in the chemoresistant cells (Figure 2G). These results were independent of histologic subtype. Notably, pGSN content was higher in chemoresistant compared to chemosensitive cells (Figure 2G). P-glycoprotein and RAB27A contents were not detectable in the sensitive cells; however, weak signals were observed in the chemoresistant cells (Figure 2G). Moreover, CDDP-induced apoptosis was significantly higher in the chemosensitive cells compared with the chemoresistant cells (Figure 2G).

### 3.3. pGSN Regulates sEV Release of CDDP and Chemoresponsiveness in OVCA Cells

**Figure 3:**
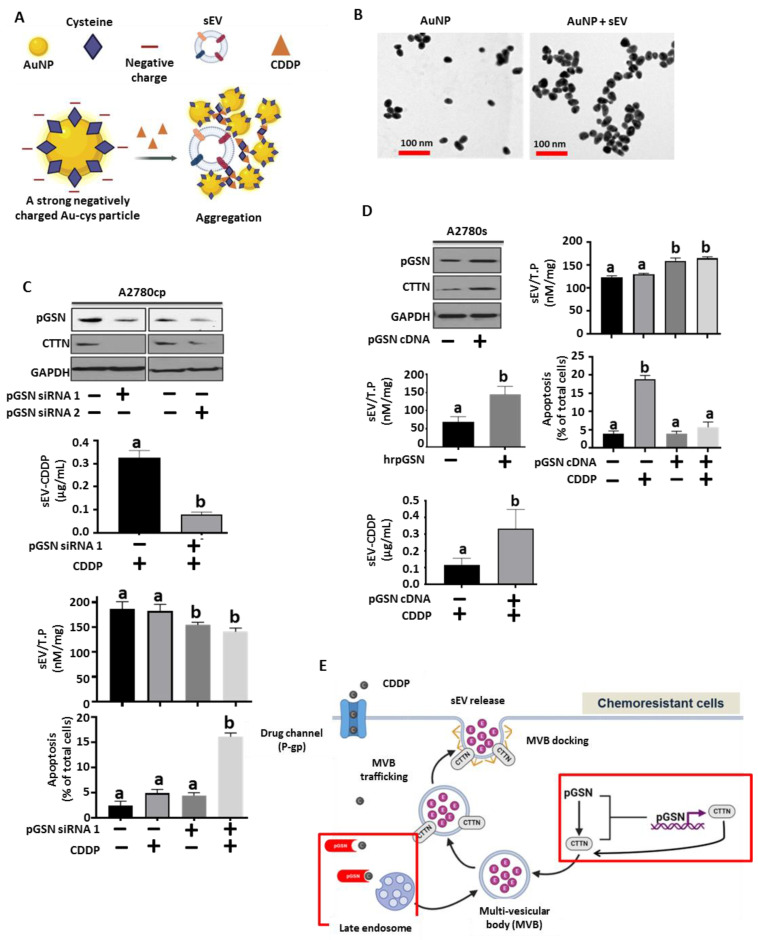
pGSN regulates sEV release of CDDP and chemoresponsiveness. pGSN downregulation in chemoresistant cells reduces CTTN content, sEV production and sEV-CDDP release whereas the opposite occurs when pGSN is over-expressed in chemosensitive cells. (**A**) Negatively charged cysteine-capped gold particles (AuNP) react with CDDP and sEVs to form aggregates which are used for the SERS quantification. The rate of aggregation is proportional to the concentrations of CDDP and sEV. (**B**). Transmission electron microscope (TEM) images of mono-stable colloid of AuNP (without sEVs; left panel) and aggregated cysteine-capped AuNP (with sEVs and CDDP; right panel). The biological components in the solution were unable to be visualized in the TEM images since the sEVs were sonicated. pGSN was (**C**) silenced in chemoresistant cells (siRNA; 50 nM, 24 h) and (**D**) over-expressed in chemosensitive cells (cDNA; 2 ug, rhpGSN; 10 μM; 24 h). Cells were then treated with or without CDDP (10 μM; 24 h). pGSN, CTTN and GAPDH contents were assessed by Western blot and cisplatin-induced cell death analysed by Hoechst staining. sEV and CDDP concentrations were determined by biosensor aggregation using Raman spectroscopy. (**E**) A hypothetical model suggesting the role of pGSN in the sEV release of CDDP from chemoresistant OVCA cells. Results are expressed as means ± SD from three independent replicate experiments. [(**A**–**C**), (a; ** *p* < 0.01 vs. b)].

The role of pGSN in sEV-mediated release of CDDP was further investigated in OVCA cells. Gold nanoparticles (AuNP) were synthesized and capped with cysteine (Au-cys) as previously demonstrated and characterized. Due to the strong negative charge of Au-cys particles, they form a mono-stable colloid in the absence of sEVs containing CDDP (Figure 3A,B). Upon the addition of sEV containing CDDP, the cysteine residues are attacked by CDDP resulting in the reduction of the surface charges of the Au-NP (Figure 3A,B). This results in Au-NP-sEV-CDDP aggregation which can be measured by surface-enhanced Raman spectroscopy (SERS); a response that is proportional to the concentration of sEV and CDDP. Mono-stable colloid formation and aggregations of the particles were confirmed using transmission electron microscopy (Figure 3B).

pGSN was silenced in chemoresistant cells using siRNAs (50 nM; 24 h; empty vector as a control) (Figure 3C) and overexpressed in chemosensitive cells using cDNA (2 μg; 24 h) and human recombinant pGSN (hrpGSN, 10 μM; 24 h) (Figure 3D and Supplementary Materials Figure S2A). pGSN, CTTN and GAPDH contents were assessed by Western blot and CDDP-induced death analyzed by Hoechst staining. SEVs were isolated and their levels together with sEV-CDDP concentrations determined using SERS. pGSN knock-down resulted in the downregulation of CTTN content; a phenomenon that decreased sEV secretion as well as sEV-CDDP content (Figure 3C and Supplementary Materials Figure S2B). Additionally, pGSN knock-down sensitized the chemoresistant cells to CDDP-induced apoptosis (Figure 3C). pGSN overexpression in chemosensitive cells increased CTTN content, sEV secretion as well as sEV-CDDP content (Figure 3D and Supplementary Materials Figure S2C). pGSN overexpression also suppressed CDDP-induced apoptosis in the chemosensitive cells (Figure 3D). Additionally, we observed that hrpGSN promoted sEV secretion in chemosensitive cells; an observation which was consistent with pGSN overexpression by cDNA (Figure 3D).

The above findings potentially suggest that pGSN regulates CTTN expression, which leads to enhanced secretion of sEVs in chemoresistant OVCA cells. Furthermore, pGSN is enriched with sulphur and metallic-binding sites; properties that might facilitate its direct binding to platinum, leading to enhanced CDDP packaging in sEVs and eventual release from the cell (Figure 3E).

### 3.4. pGSN-Mediated Release of sEV-CDDP Is Associated with the Formation of Extranuclear and Extracellular Electron-Dense Granules

**Figure 4:**
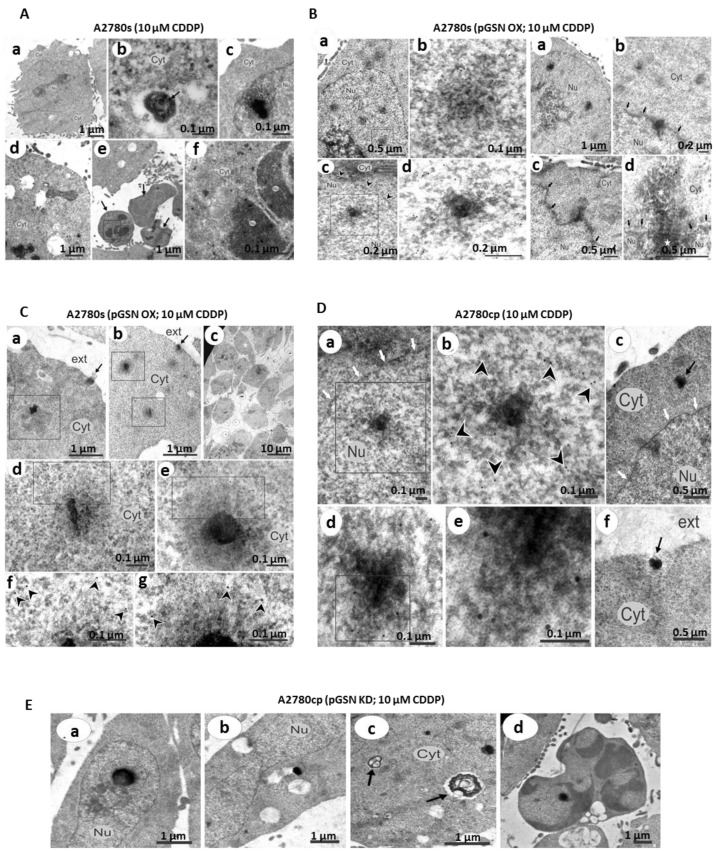
pGSN-mediated release of sEV-CDDP is associated with the formation of extranuclear and extracellular electron-dense granules. (**A**) Chemosensitive cells (A2780s) were treated with 10 μM CDDP for 24 h. A general view of the cell whose cytoplasm is filled with small electron-dense particles (panel a). Autophagic vacuole which contains an electron-dense remnant body containing small electron-dense particles (particle sample depicted by arrow) (panel b). A cell whose nucleus contains an aggregation of electro-dense particles and compact electron-dense granule (panel c). A mitochondrion in close contact with electron-light vacuole (panel d). The apoptotic bodies (indicated by arrows) of the cell that underwent collapse by apoptosis (panel e). Enlarged image of the area shown by square in the previous image; note small electron-dense particles (panel f). Nu—nucleus, Cyt—cytoplasm. Scale bar = 1 µm (panels a, d, e), 0.1 µm (panel b), 0.5 µm (panels c, f). (**B**) Chemosensitive cells (A2780s) were overexpressed with pGSN and then treated with 10 μM CDDP for 24 h. Electron-dense aggregates in the nucleus (nu) (left panel a). Enlarged image of the nuclear aggregate shown by square in panel a (left panel b). Intranuclear aggregate bearing peculiar compact electron-dense central granule (left panel c). Enlarged image of the nuclear aggregate shown by square at the panel c (left panel d). Arrowheads show nuclear membrane. The circles show colloidal gold particles labeling pGSN. Scale bar = 0.5 µm (left panel a), 0.1 µm (left panel b), 0.2 µm (left panels c, d). Mature electron-dense bodies in the central areas of the nucleus (right panel a). Mature electron-dense body in close contact with nuclear membrane (shown by arrows; right panel b). Electron-dense substance protruding from the nuclear membrane to the cytoplasm (Cyt; right panel c). pGSN staining on the periphery of the intranuclear electron-dense body (marked by star), on the nuclear membrane (showed by square) and in some areas of the cytoplasmic friable substance (right panel d). The circles show colloidal gold particles labeling pGSN. Scale bar = 1 µm (right panel a), 0.2 µm (right panel b), 0.5 µm (right panel c, d). (**C**) Electron-dense bodies in the cytoplasm (Cyt; panels a, b). Electron-dense bodies (shown by circles) in the extracellular space (panel c). Gradually enlarging projections of the electron-dense enlarged from panels a and b (panels d–g). Arrowheads show pGSN staining. Ext—extracellular space. Scale bar = 1 µm (panels a, b), 10 µm (panels c), 0.1 µm (panels d–g). (**D**) Chemoresistant cells (A2780cp) were treated with 10 μM CDDP for 24 h. Electron-dense substance located in the nucleus (panels a, b; note the peripheral staining of pGSN in panel b). Electron-dense substance undergoing penetration from the nucleus (Nu) to the cytoplasm (Cyt) through the nuclear membrane (shown by arrows) and cytoplasmic granule (shown by arrow) (panel c). Gradually enlarging cytoplasmic electron-dense granule with peripheral pGSN staining (panels d, e). Electron-dense granule (shown by arrow) which is undergoing exocytosis. Scale bar = 0.1 µm (panels a, b), 0.5 µm (panel c), 0.1 µm (panels d, e), 0.5 µm (panel f). (**E**) Chemoresistant cells with pGSN knock down were treated with 10 μM CDDP for 24 h. Nuclear electron-dense body (panel a). Cytoplasmic electron-dense [panel b; note close contact with electron-light vesicles]. Autophagic vacuoles containing the electron-dense remnant bodies (showed by arrows; panel c). Apoptotic cell with electron-dense material (shown by circle; panel d). Scale bar = 1 µm (panels a–d).

To understand the interaction between pGSN, sEV and CDDP secretion at the cellular level, electron microscopy was used to study the ultra-structural basis of sEV-CDDP release of OVCA cells when pGSN is manipulated. pGSN in chemoresistant cells was silenced using siRNAs (50 nM; 24 h; empty vector as control) and overexpressed in chemosensitive cells using cDNA (2 μg; 24 h). The cells with pGSN manipulation were then treated with CDDP (10 μM; 24 h). Cells were collected and processed for iEM (pGSN staining in sEVs and multivesicular bodies) and TEM (ultra-structural analyses) (Figure 4). In chemosensitive cells treated with CDDP, there were electron-dense granules in the cytoplasm and the nucleus. Moreover, there were cells that underwent collapse by apoptosis as well as apoptotic bodies containing dense granules in both the cytoplasm and parts of fragmented nucleus. The upregulation of pGSN in chemosensitive cells resulted in the amalgamation of dense granules in the nucleus. The enlarged image of these aggregates revealed pGSN staining situated in the area of the dense granules. A more dispersed peripheral substance is observed surrounding the dense central granules and enlarging the mature aggregate revealed that pGSN staining mostly situated in the peripheral substance rather than in the central granules. The dense granules seem to be formed in the nucleus and move through the nuclear membrane into the cytoplasmic area. We also observe the staining of pGSN on the periphery of the intranuclear dense-granules, nuclear membrane and in some areas of the cytoplasmic granules, suggesting the possible involvement of pGSN in the process. We also observed the dense granules close to the plasma membrane and exiting the cell in the intercellular space via exocytosis. An enlargement of the granules showed the localization of pGSN on the periphery of the collar surrounding the granules suggesting a possible role of pGSN in granule formation and secretion.

Chemoresistant cells treated with CDDP showed electron-dense granules located in the nucleus, penetrating through the nuclear membrane and being released into the intercellular space via exocytosis. Enlarging the dense granules reveals pGSN staining at the periphery of the dense structures, suggesting the potential role of pGSN in the formation and extranuclear transportation of the dense structures. When pGSN was knocked down in the chemoresistant cells, an opposite phenomenon was observed. Although the dense granules were present in the nucleus and cytoplasm, these structures were not secreted. Apoptotic cells were observed containing electron-dense granules. These suggest that decreased pGSN levels attenuate the extranuclear transportation of dense granules, triggering apoptosis. Corresponding details on individual panels are provided in the figure legend.

### 3.5. pGSN Positively Correlates with Dense-Granule-Related Proteins and Are Associated with Chemoresistance

We further investigated how sEV secretion relates with the dense granules release from the cells. In the chemosensitive cells, sEV containing pGSN are spotted in the multivesicular bodies (MVB) (Supplementary Materials Figure S3A). Upregulating pGSN expression in the chemoresistant cells resulted in the penetration of the dense granules into the MVBs and their subsequent secretion via exocytosis (Supplementary Materials Figure S3B), suggesting that MVB might be the potential vehicle transporting the dense granules. A similar phenomenon was observed in the plasma-derived sEVs of a chemoresistant OVCA patient (Supplementary Materials Figure S3C).

To determine whether pGSN is associated with the dense granule formation, we interrogated the ovarian cancer TCGA public dataset (http://gepia.cancer-pku.cn/index.html) accessed on 12 February 2022 to assess the correlation (Spearman coefficient) between pGSN and genes (BLOC1S1, BLOC1S2, BLOC1S3, BLOC1S4, BLOC1S5, BLOC1S6, DTNBP1, RAB32, RAB38, SNAPIN, VPS11, VPS16, VPS18, VPS33A, VPS33B, VPS39, VPS41) associated with dense granule formation. Except for BLOC1S1, BLOC1S2 and RAB38 genes, pGSN positively correlated with all the genes with the strongest being VPS18, VPS39 and VPS41 (Table S2). Additionally, we interrogated ovarian cancer datasets (http://www.rocplot.org/ovarian/index, accessed on 12 February 2022) to determine the differential expression (box plot; chemoresistance vs. chemosensitive) and test performance (receiver operating characteristic (ROC) analysis; chemoresistance prediction) of VPS18, VPS39 and VPS41. We found that the three genes were upregulated in chemoresistant OVCA patients compared with their sensitive counterparts and exhibited a significant prediction of chemoresistance (Figure S3D–F). This supports the hypothesis that pGSN might potentially be involved in the formation of dense granules by regulating VPS18, VPS39 and VPS41.

### 3.6. SEV to CA125 Ratio (sEV/CA125) Is a Strong Predictor of Stage 1 Disease, Chemoresponsiveness and Favorable Survival Outcomes in OVCA Patients

**Figure 5:**
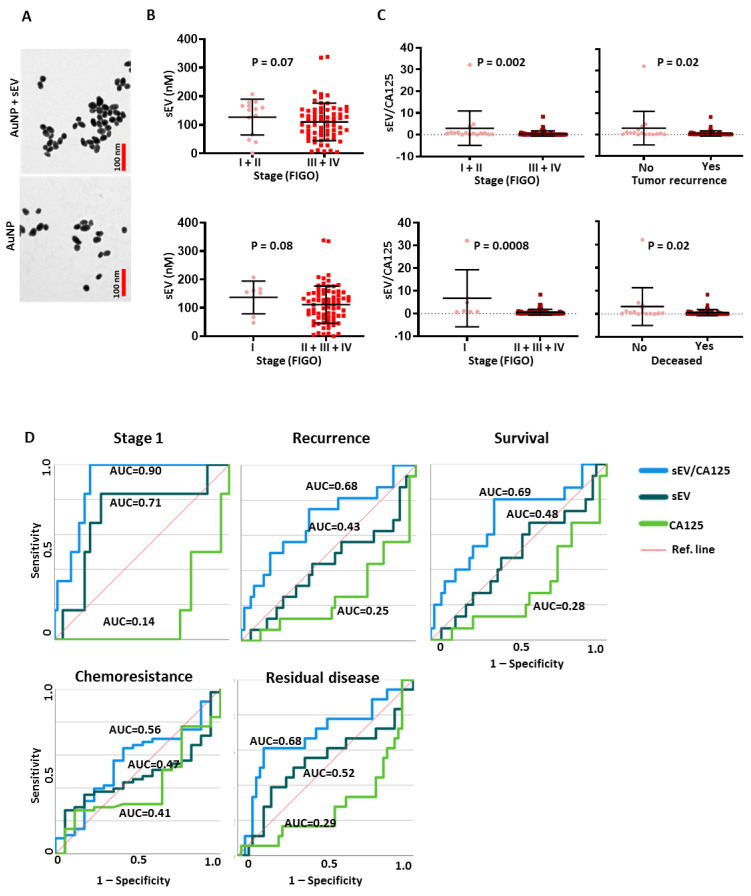
sEV/CA125 predicts stage 1 and tumor recurrence with high test accuracy. (**A**) Transmission electron microscope (TEM) images of mono-stable colloid of AuNP (without plasma-derived sEVs; left panel) and aggregated cysteine-capped AuNP (with plasma-derived sEVs; right panel). The biological components in the solution did not appear visible in the TEM images. SEVs were isolated from the plasma of human OVCA patient with pre-determined CA125, sEV or sEV/CA125 were correlated with patient clinical outcomes. (**B**) The mean levels of sEV concentrations were compared between early (I + II) and late (III + IV) stages as well as stage I and >stage 1 groups. Patients with late stage OVCA had lower levels of sEV concentration compared with early stage or stage 1 patients. (**C**) The mean levels of sEV/CA125 were correlated with OVCA clinical outcomes (stages, tumor recurrence and survival). (**D**) The test performances of sEV, sEV/CA125 and CA125 in the prediction of stage 1, tumor recurrence, survival, chemoresistance and residual disease were compared using ROC curves.

After characterizing our biosensor in OVCA cell lines, we extended its application to measuring SEV concentration in human patient plasma. Plasma samples were collected from 99 OVCA patients with HGS, LGS and unverified histologic subtypes (Table S1). Plasma-derived sEVs from OVCA patients and non-OVCA subjects were isolated and characterized (Figure 1). The biosensor application was tested with the buffers used in the sEV isolation to ensure they do not interfere with the SERS measurement (Figure S4A,B). Although the buffers increased the aggregation of the nanogold particles, this could easily be normalized and did not affect CDDP quantification (Figure 4B,C). Mono-stable colloid formation and Au-NP aggregation with the plasma-derived sEV were confirmed using transmission electron microscopy before SERS quantification was performed (Figure 5A).

**Figure 6:**
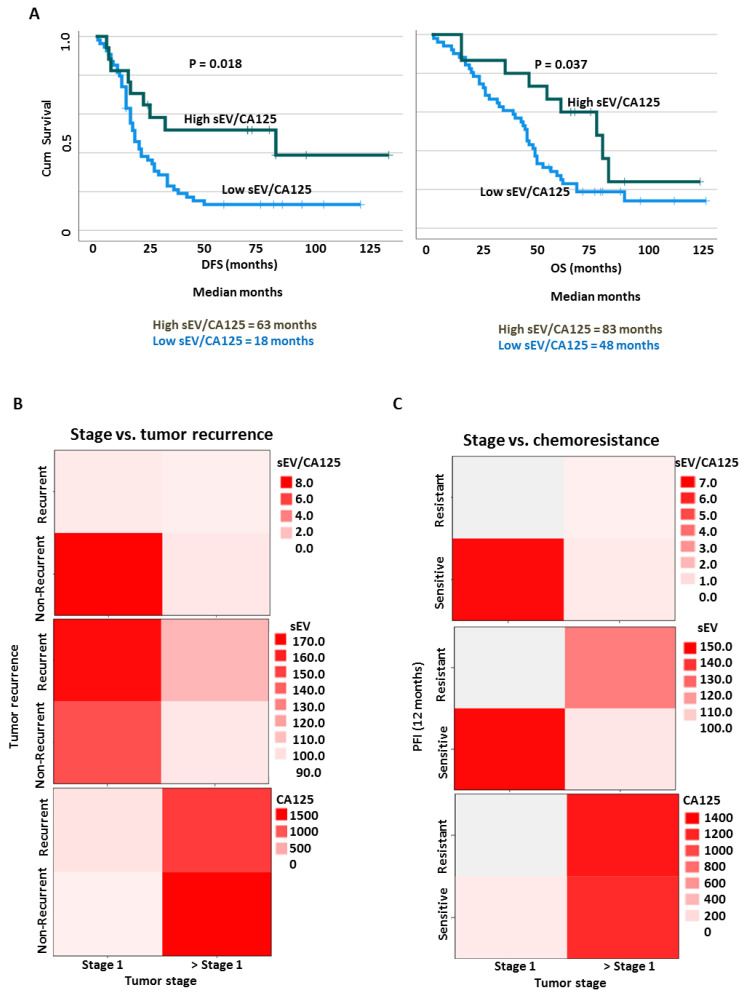
(**A**–**C**) High levels of sEV/CA125 provide favorable survival outcomes to OVCA patients. sEV/CA125 levels in OVCA patients (N = 99) were correlated with progression-free survival (PFS) and overall survival (OS). Kaplan–Meier survival curves with dichotomized sEV/CA125 levels (low and high groups, cut-off = 68.8) and log rank test were used to compare the survival distributions between the groups. N = number of patients. (**B**) sEV/CA125 predicts more than one clinical outcome of OVCA. SEV/CA125, sEV and CA125 were used in heat map analyses to predict stage 1 and tumor recurrence as well as stage 1 and chemoresistance.

The sEV concentration and EV/CA125 ratio were determined and their mean ± SD values correlated with patient clinical outcomes (stage, tumor recurrence, survival, chemoresistance, and residual disease) (Figure 5, Figure 6 and Figure S5). We observed that patients with advanced stages of OVCA had levels of sEVs comparable to those in early stages (Figure 5B); however, no significant difference in sEV concentrations was observed among the patients and the healthy control individuals. There was also no correlation detected with the presence of residual disease (RD < 1 cm vs. RD > 1 cm), chemoresistance (PFI < 6 mo vs. PFI > 6 mo), tumor recurrence (yes vs. no), and the level of CA125 (low vs. high) (Supplementary Materials Figure S5A). SEVs had a negative correlation with CA125, hence we developed a ratio with both markers to investigate their clinical utility (Figure S5B). SEV/CA125 ratio had better clinical utility compared to sEV and CA125 concentrations alone with regard to OVCA stage, tumor recurrence and disease state (Figure 5C). However, a trend (although not significant) was observed with residual disease and histologic differentiation subtypes (Figure S5C). Using ROC analysis, we determined the test performances of sEV, sEV/CA125, and CA125 in predicting patient clinical outcomes (Figure 5D). We found that sEV/CA125 was the most robust in predicting stage 1 disease, non-recurrence, patient survival, chemoresponsiveness and optimal residual disease in OVCA patients (Figure 5D).

Kaplan–Meier survival curves with dichotomized sEV/CA125 ratio (low vs. high, cut-off = 68.8) and log rank test were used to compare the survival distributions between the groups (Figure 6A). We observed that patients with high sEV/CA125 had significantly improved disease-free survival (DFS; 63 months vs. 18 months, *p* = 0.018) as well as overall survival (OS; 83 months vs. 48 months, *p* = 0.037) compared with patients with low sEV/CA125 (Figure 6A). We further investigated whether sEV/CA125 could be used to predict multiple clinical outcomes using a heat map (Figure 6B,C). Compared with sEV and CA125 individually, sEV/CA125 ratio provided a significant prediction value for multiple clinical outcomes. Patients with increased sEV/CA125 were mostly in stage 1 and did not develop recurrence or resistance to treatment (Figure 6B,C). SEV and CA125 concentrations alone were not predictive of clinical outcomes.

## 4. Discussion

For the first time, we have shown evidence that pGSN plays a regulatory role in sEV secretion as well as sEV-mediated release of CDDP; factors that are involved in OVCA chemoresistance. Additionally, we extended the application of our novel sEV-based biosensor to a small OVCA patient cohort to develop a highly sensitive diagnostic platform for the detection of early stage OVCA and prediction of chemoresponsiveness.

Drug efflux is a key determinant of chemoresistance in many cancer types, including OVCA [34,35,36,37]. Most studies have identified multi-drug resistant proteins such as p-glycoprotein to be responsible for drug efflux in cancer cells, thus rendering chemotherapeutic agents unable to induce death in cancer cells [36,37]. In this present study, we have demonstrated that sEVs serve as a vehicle for exporting chemotherapeutic agents from OVCA cells rather than using drug transport channels. Chemoresistant OVCA cells treated with CDDP secreted an increased amount of sEVs as well as sEV containing CDDP compared with their sensitive counterparts. This could potentially explain why targeting drug transport channels, such as P-glycoprotein, has not yielded any therapeutic benefit in OVCA given the role of sEVs in drug efflux. This suggests that sEV secretion is a potential therapeutic target to tackle chemoresistance in OVCA.

Targeting sEV secretion also demands an understanding of the mechanism underlying the increased secretion of sEVs and sEV-CDDP from chemoresistant cells. pGSN is overexpressed in chemoresistant OVCA compared with chemosensitive cells and transported via sEVs [10]. The positive association between pGSN and sEVs motivated us to investigate the role of pGSN in sEV secretion. We observed that silencing pGSN reduced the expression of CTTN (involved is sEV-release mechanisms) in chemoresistant cells resulting in decreased sEV secretion and EV-CDDP release. The opposite occurred when pGSN was overexpressed in chemosensitive OVCA cells. Targeting pGSN in the chemoresistant cells resulted in increased accumulation of CDDP leading to increased apoptosis, suggesting that pGSN is a key regulator of CTTN expression. These findings are consistent with those of a previous study in which pGSN and CTTN were found to be upregulated and co-localized in resistant pancreatic cancer cell lines [38]. Furthermore, recent studies have shown that proteins rich in sulphur and amines have high affinity for cisplatin binding [39,40]. Since pGSN has these properties as well as more than 10 metal-binding sites, there is a possibility that it could directly bind to CDDP and facilitate their packaging into sEVs for secretion. This will be worth investigating in the future.

Dense granule biogenesis originates from early and late endosomes with MVBs serving as likely precursors [41,42]. Their biogenesis is tightly controlled by protein sorting and regulating machineries such as BLOC1S1, BLOC1S2, BLOC1S3, BLOC1S4, BLOC1S5, BLOC1S6, DTNBP1, RAB32, RAB38, SNAPIN, VPS11, VPS16, VPS18, VPS33A, VPS33B, VPS39 and VPS41 [41,42]. Dense granules are abundant in ADP, ATP, calcium, pyrophosphate and serotonin, which are key molecules involved in platelet activation and function [41,42]. To date, there has been no study on the role of dense granules outside platelet function as well as in cancer pathogenesis. For the first time, we observed that dense granules are critical players in OVCA chemoresistance. In chemoresistant condition, pGSN is upregulated which prevents cells from undergoing CDDP-induced death. Interestingly, we found that increased pGSN resulted in the formation of nuclear dense granules which are transported into the cytoplasm and later released out of the cell through exocytosis. This phenomenon only happens when chemoresistant cells are treated with CDDP, suggesting that dense granules could facilitate the efflux of CDDP out of the nucleus to prevent DNA damage and cell death. Although these dense granules are formed in the sensitive cells when treated with CDDP, they are unable to be released out of the cell which was evident by apoptotic bodies containing electron-dense remnant granules in cells that had undergone apoptosis. pGSN positively correlates with dense-granule-associated proteins and present at the periphery of the nuclear and cytoplasmic dense structures as well as the nuclear membrane. This possibly suggests that pGSN might be involved in the biogenesis and transportation of these granules, presenting a novel survival mechanism by which cancer cells secrete chemotherapeutic agents. Targeting pGSN will potentially result in the attenuation of dense granule formation and transportation, thus accumulating intracellular CDDP leading to cancer cell death. What remains to be determined is whether pGSN serves as a transcription factor for the dense-granule-associated proteins or not. Furthermore, it is worth investigating whether MVBs serve as precursors for the biogenesis of dense granules in OVCA chemoresistance or the granules fuse with MVBs in order to transfer their content (pGSN and CDDP) to the sEVs.

Late diagnosis poses a huge challenge to therapeutic success and patient survival. Developing a highly sensitive diagnostic platform for early OVCA diagnosis is of urgent need. With heterogeneity and protein interference as major challenges when using SERS for sEV analysis, we have developed a biosensor to overcome this challenge. This biosensor can react with sEVs and CDDP simultaneously; a strategy that enables us to quantify both sEVs and CDDP simultaneously using SERS. This strategy is highly sensitive and superior to those of other studies that have developed gold nanoparticles for sEV analysis alone.

We examined the diagnostic utility of our sEV-based biosensor in a small pre-operative OVCA cohort. Although sEVs could be detected and quantified, their diagnostic value was not as strong as when combined with CA125. This could potentially be due to the fact that the biosensor recognized sEVs from origins other than OVCA. To overcome this, we used a ratio of sEVs to CA125. We observed that the sEV/CA125 ratio had a significantly improved diagnostic value. sEV/CA125 was significantly elevated in stage 1 patients compared to stages 2–4 and outperformed CA125 and sEV in identifying stage 1 disease. This is a promising marker given that conventional biomarkers have only provided modest diagnostic value. Interestingly, sEV/CA125 also outperformed CA125 and sEVs in predicting tumor recurrence, chemoresistance, residual disease and patient survival. This is an exciting finding given that there is currently no reliable biomarker to predict tumor recurrence and chemoresistance. OVCA patients with increased sEV/CA125 had prolonged survival compared with those with low sEV/CA125, suggesting that sEV/CA125 could be used as a potential marker for early stage OVCA and for predicting patient survival outcomes prior treatment or surgery. With limited biomarker options for early stage OVCA and prediction of chemoresistance, sEV/CA125 could be further investigated to provide the necessary diagnostic tool required.

## 5. Conclusions

We have demonstrated that pGSN regulates sEV and sEV-CDDP secretions via CTTN upregulation leading to decreased intracellular CDDP accumulation and apoptosis. These processes further suppress chemo-responsiveness in OVCA cells. Additionally, we applied a novel biosensor to develop a platform that is effective in identifying stage 1 disease and predicting tumor recurrence and chemoresistance. Although these findings are promising and bear important clinical relevance, further validation of the platform is needed in a larger patient cohort and animal models. Moreover, due to the lower volumes of our plasma samples, ExoQuick Ultra presented as the suitable isolation method for our sEVs. Thus, we acknowledge that our sEVs could have larger size distribution as well as minimal contamination with protein aggregates; limitations we hope to overcome in our ongoing studies where large sample volumes are being collected.

## Data Availability

The authors confirm that the data supporting the findings of this study are available in the article and/or presented as supplementary information. Any other information will be available on reasonable request from the corresponding author.

## Supplementary Materials

The following supporting information can be downloaded at: https://www.mdpi.com/article/10.3390/cancers15092566/s1, Figure S1: OVCA tumor expressions of drug transport proteins and RAB27A; Figure S2: pGSN regulates CDDP and exosome secretion in HGS OVCA cells; Figure S3: pGSN positively correlates with dense granules-related proteins and are associated with chemoresistance; Figure S4: Validation of biosensor on human OVCA plasma-derived EVs. Figure S5: Prognostic significance of exosome and Ex/CA125 in OVCA patients; Figure S6: Original, uncropped Western blot membrane; Table S1: Characteristics of patients and subjects; Table S2: Correlation between GSN and dense granules-associated genes; Table S3: Information on antibodies and reagents; Table S4: Information on OVCA cell lines; Table S5: Customized siRNA oligonucleotide duplexes. References [43,44,45,46,47,48] are cited in the supplementary materials.
