# Supplementary material for: The Application of an Extracellular Vesicle-Based Biosensor in Early Diagnosis and Prediction of Chemoresponsiveness in Ovarian Cancer
Source: Cancers (Basel). 2023 Apr 30;15(9):2566. doi: 10.3390/cancers15092566 (PMC10177169; doi:10.3390/cancers15092566)
Supplement: Supplementary file 1 [file cancers-15-02566-s001.zip › cancers-2280513-supplementary.pdf]

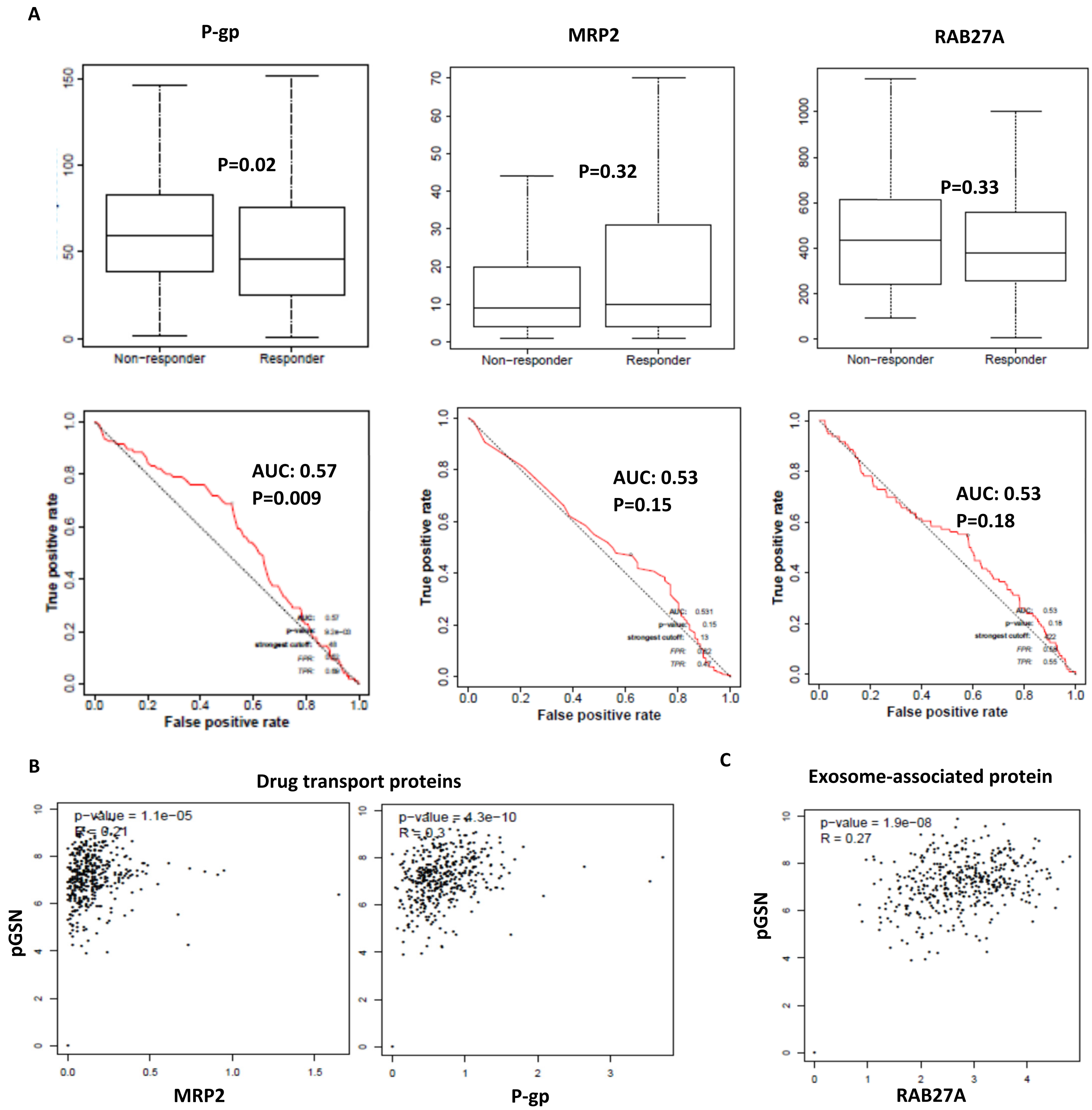

Figure S1. OVCA tumor expressions of drug transport proteins and RAB27A. Correlation between pGSN and drug transport proteins as well as exosome-associated proteins in OVCA patient tumors. (A) Our interrogation of TCGA public dataset revealed that P-gp but not MRP2 and RAB27A are significantly predictive of chemoresistance in human OVCA tumors. (B) A positive correlation was observed between pGSN and MRP2, P-gp and (C) RAB27A. N = 958.

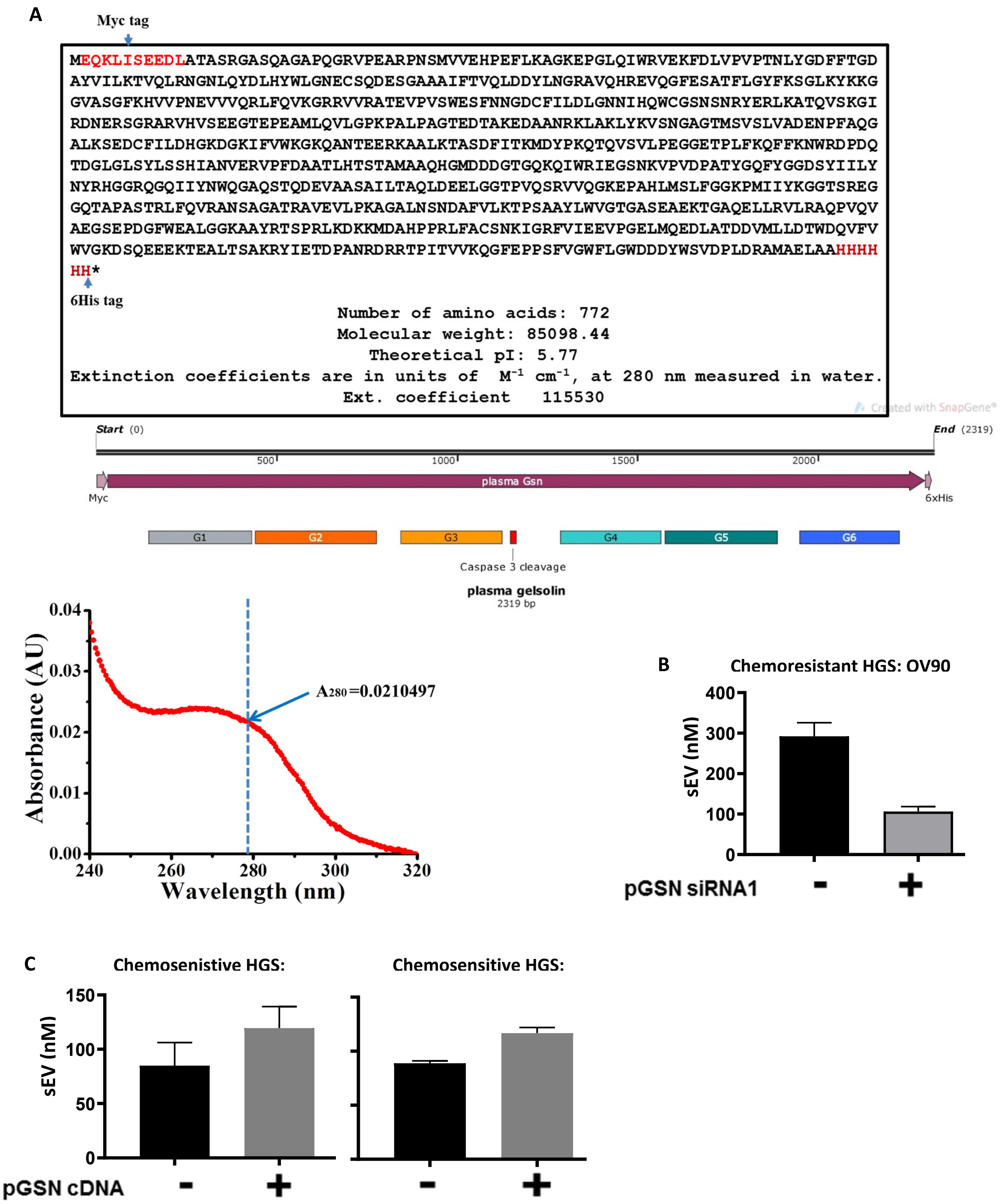

Figure S2. pGSN regulates exosomal release in high grade serous (HGS) OVCA cell lines. (A) Human recombinant pGSN was synthesized with Myc and 6His tags inserted. The refolded gel UV absorbance measured with A280=0.0210497. pGSN downregulation in chemoresistant HGS cells reduced sEV production whereas the vice-versa occurs when pGSN in over-expressed in chemosensitive HGS cells. pGSN was (B) silenced in chemoresistant cells (siRNA; 50 nM, 24 h) and (C) over-expressed in chemosensitive cells (cDNA; 2 ug, 24 h). Small EV concentrations were determined by biosensor-sEV aggregation using Raman spectroscopy. Results are expressed as means ± SD from three independent replicate experiments.

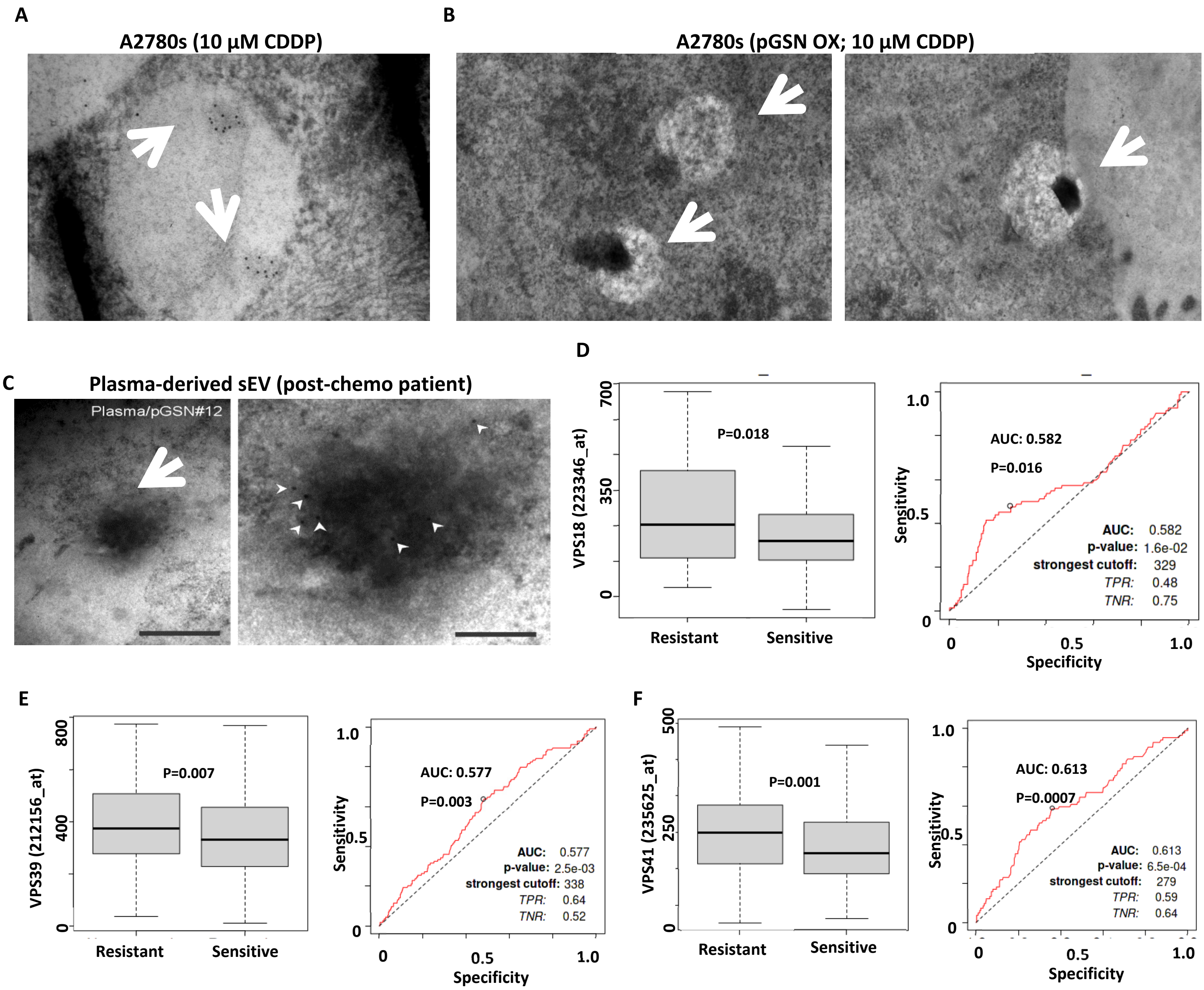

Figure S3. pGSN positively correlates with dense granules-related proteins and are associated with chemoresistance. (A) Chemosensitive cells were treated with 10 μM for 24 h. Multivesicular bodies with pGSN staining identified in the cytoplasm. (B) Chemosensitive cells overexpressed with pGSN were treated with 10 μM for 24 h. Arising of dense granules in the multivesicular bodies (left panel). Exocytosis of multivesicular body with dense granules. (C) Plasma-derived sEVs were isolated from a post-chemo OVCA patient. Identification of pGSN with dense granules. (D-F) Our investigation of TCGA public dataset revealed that VPS18, VPS39 and VPS41 are upregulated in chemoresistant patients compared with their sensitive counterparts. The test performances VPS18, VPS39 and VPS41 were evaluated using ROC curves and significant predictions of chemoresistance were observed.

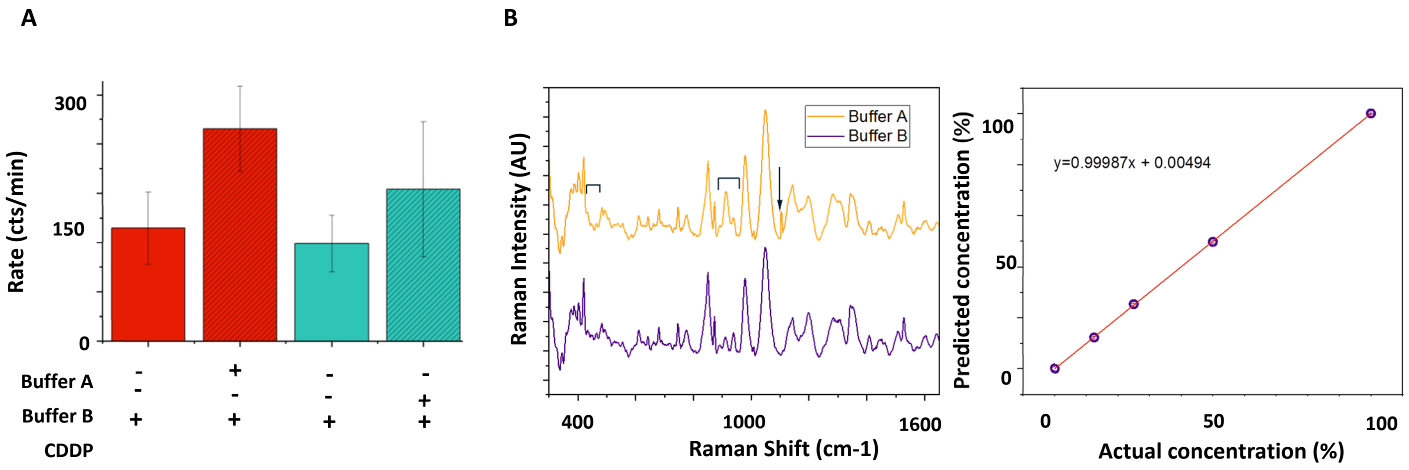

Figure S4. Validation of biosensor on human OVCA plasma-derived Evs. Plasma-derived small EV isolation, characterization and biosensor detection. (A) Biosensor detection of small EVs and CDDP were performed using buffer A and B from the Exoquick Ultra kit. (B) Raman peaks of Buffer A and B were generated to determine their composition and potential influence on exosome and CDDP detection.

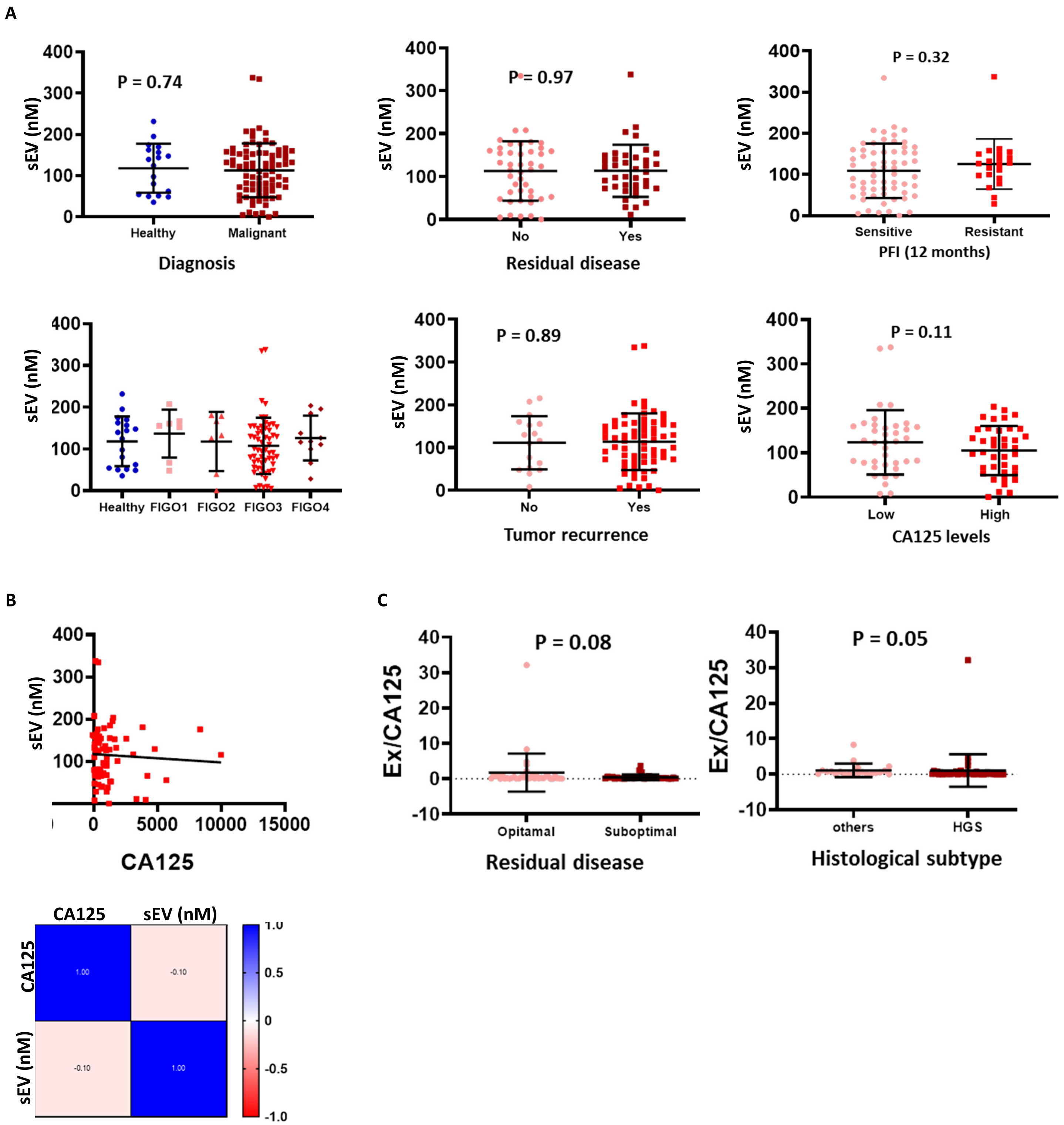

Figure S5. Prognostic significance of sEVs and sEVs/CA125 in OVCA outcomes. Small EVs were isolated from the plasma of human OVCA patient with pre-determined CA125. (A) Mean ± SD of sEV or sEV/CA125 were determined and compared within clinical outcomes (diagnosis, residual disease, chemoresistance, stage, tumor recurrence and CA125 levels). (B) Plasma-derived sEVs were correlated with CA125 in malignant OVCA patients. (C) The mean ± SD levels of sEV/CA125 were determined and compared within OVCA clinical outcomes (residual disease and histological sub-type).

Figure 1

D

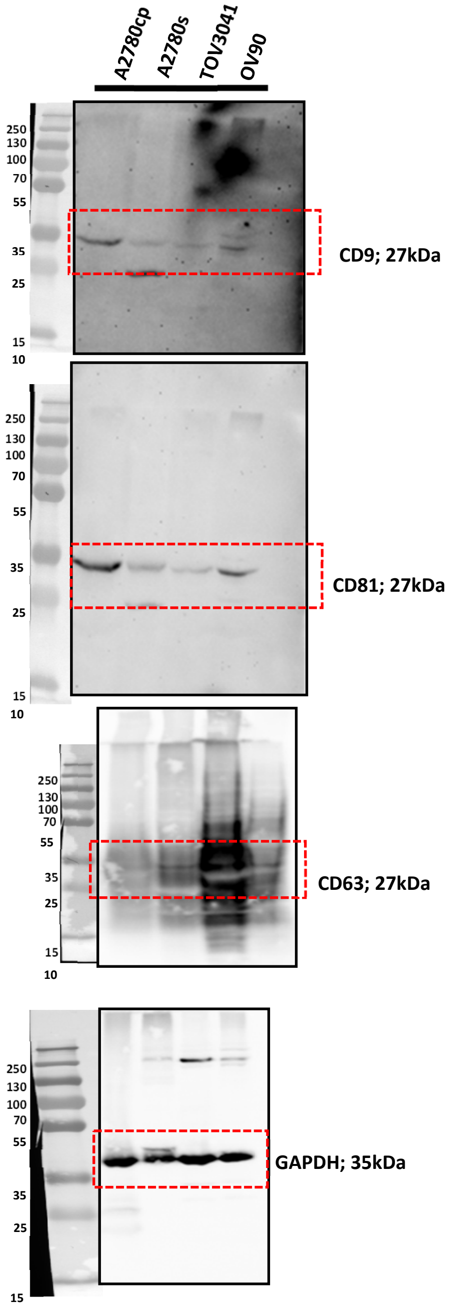

cd9	Intensity		cd81	Intensity
A278cp	14.483		A278cp	45.951
A280s	4.038		A280s	15.764
TOV3041	25.389		TOV3041	10.905
OV90	42.408		OV90	22.176
cd63	Intensity		gapdh	Intensity
A278cp	76.024		A278cp	131.461
A280s	118.784		A280s	108.39
TOV3041	198.507		TOV3041	125.362
OV90	106.348		OV90	103.415

G

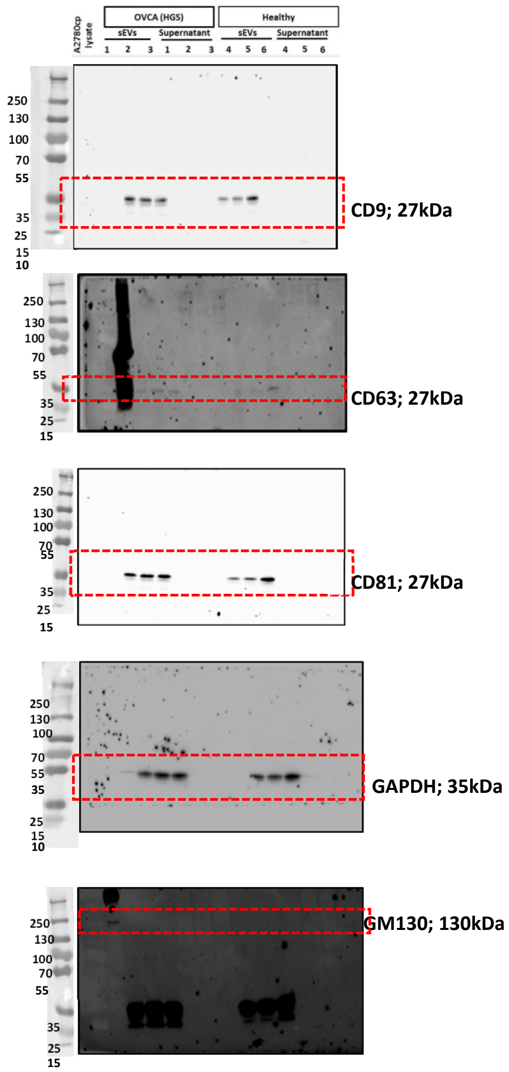

cd9	cd63	cd81	gapdh	gm130
0	113.603	0	2.779	4.294
30.957	8.767	53.582	35.019	0
41.788	11.014	68.207	50.744	0
33.953	5.757	70.266	48.289	0
0	0	0	0	0
0	0	0	0	0
0	0	0	0	0
14.447	3.938	23.128	26.78	0
18.773	3.729	31.819	35.672	0
40.837	11.42	81.625	55.849	0
0	0	0	0	0
0	0	0	0	0
0	0	0	0	0

Figure 2

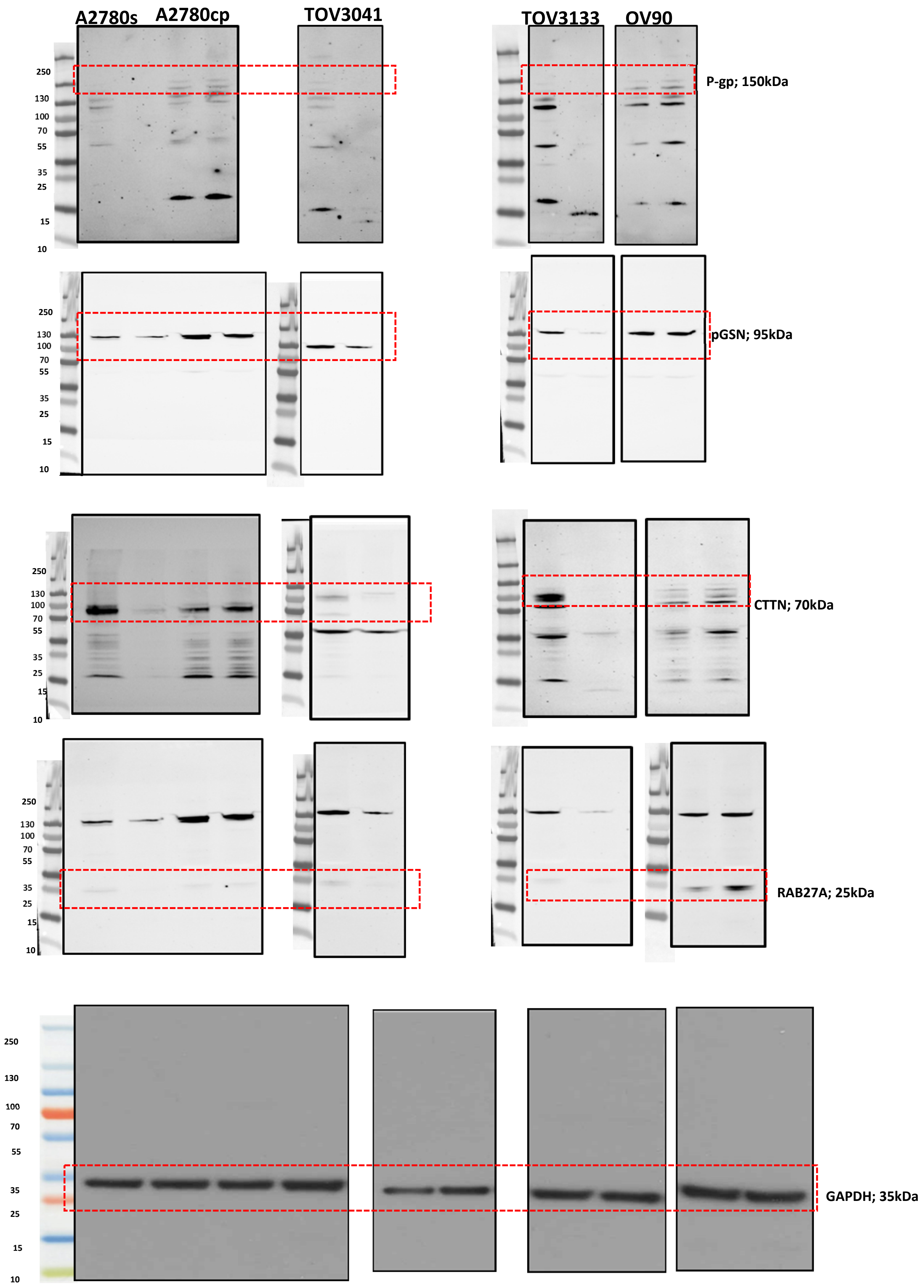
A2780s
A2780cp
TOV3041
TOV3133
OV90
250
130
100
70
55
35
25
15
10
P-gp; 150kDa
pGSN; 95kDa
CTTN; 70kDa
RAB27A; 25kDa
GAPDH; 35kDa

Figure 2. Densitometries.

	P-gp	pGSN	CTTN	RAB27A
**A2780s**	0.081568	0.651509	1.538458	0.139146
	-0.04528	0.322574	0.059514	0.021438
**A2780cp**	0.129112	1.459527	0.521966	0.058488
	0.201257	0.940696	0.636908	0.080821
**TOV3041G**	0.022972	1.263392	0.492793	0.225417
	0	0.500036	0.083264	0.072978
**TOV3313**	0.062409	0.386841	1.705355	0.063532
	0	0.036592	0.01263	0.021836
**OV90**	0.160447	0.513812	0.267786	0.269868
	0.327633	0.619517	0.590105	0.894564

Figure 3

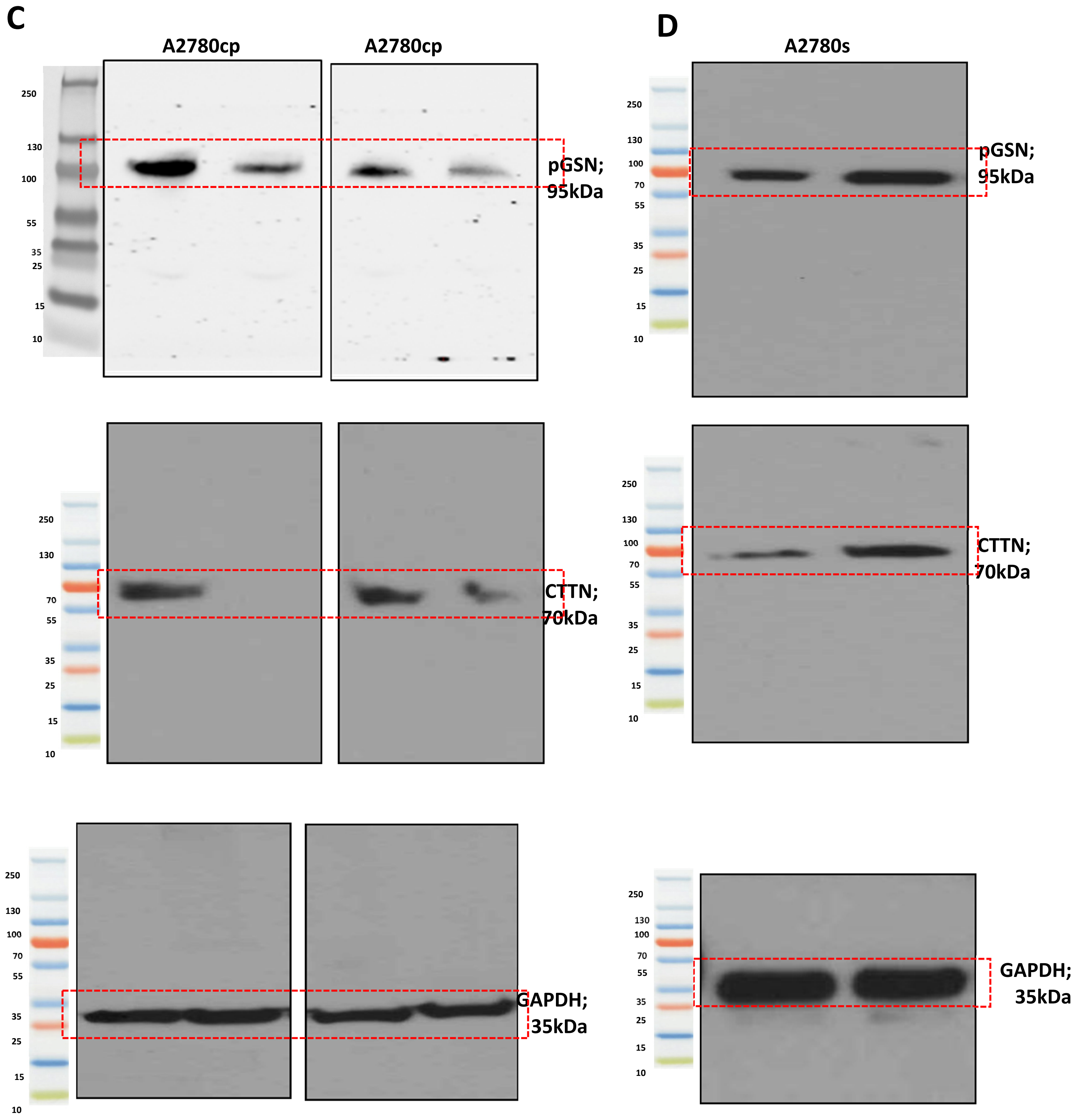

	pGSN	CTTN				pGSN	CTTN
**siRNA 1**	1.529461	0.743989			**cDNA**	0.421660949	0.164514
	0.604649	0.01216				0.791095521	0.466701
**siRNA 2**	0.710532	0.784277					
	0.365677	0.334077					

Figure S6. Original, uncropped Western blot membrane.

Table S1. Characteristics of patients and subjects.

Variable	Number of Patients
**Age (Range; 36 – 82 years)**	
≤61	51
>61	48
**Stage (FIGO)**	
1	10
2	11
3	67
4	11
**Histological Subtypes**	
Not verified	26
High Grade Serous (HGS)	69
Low Grade Serous (LGS)	4
**Residual disease (RD)**	
≤1 cm	50
>1 cm	42
**Healthy Subjects**	20

FIGO, International Federation of Gynecology and Obstetrics.

Table S2. Correlation between GSN and dense granules-associated genes.

Gene	Spearman's Test	P-value
**BLOC1S1**	-0.21	1.5e-05
**BLOC1S2**	0.034	0.49
**BLOC1S3**	0.25	1.2e-07
**BLOC1S4**	0.23	1.2e-06
**BLOC1S5**	0.21	2e-05
**BLOC1S6**	0.23	1.1e-06
**DTNBP1**	0.2	4e-05
**RAB32**	0.13	5.6e-03
**RAB38**	0.0025	0.96
**SNAPIN**	0.017	0.72
**VPS11**	0.28	3.4e-09
**VPS16**	0.19	1.1e-04
**VPS18**	0.44	1e-21
**VPS33A**	0.16	8.4e-04
**VPS33B**	0.16	7e-04
**VPS39**	0.38	4.6e-16
**VPS41**	0.36	3.4e-14

Table S3. Information on antibodies and reagents.

Primary Antibodies/Reagent						Secondary Antibodies					
**Application**	**Target**	**Reagent**	**Company**	**Catalog #**	**Dilution**	**Antibody**	**Conjugate**	**Company**	**Catalog #**	**Dilution**	**Note**
**WB**	pGSN	Anti-pGSN Goat polyclonal	Antibodies online(Atlanta, USA)	ABIN1019662	1:1000	Dnk polyclonal to Goat IgG	HRP	Abcam (Toronto, Canada)	Ab97110	1:2000	
**WB**	GAPDH	Anti-actin mouse monoclonal	Abcam (Toronto, Canada)	ab8226	1:1000	Goat Anti-mouse IgG (H+L)	HRP	Bio-Rad (Mississauga, Canada)	170-6516	1:2000	
**WB**	pGSN	Anti-pGSN mouse polyclonal	Antibodies online(Atlanta, USA)	ABIN659182	1:500	Goat Anti-mouse IgG (H+L)	HRP	Bio-Rad (Mississauga, Canada)	170-6516	1:2000	
**WB**	CD9	Anti-CD9 rabbit	System Biosciences	EXOAB-CD9A-1	1:1000	Goat Anti-rabbit IgG (H+L)	HRP	Bio-Rad (Mississauga, Canada)	1706515	1:2000	
**WB**	CD63	Anti-CD63 mouse monoclonal	Abcam (Toronto, Canada)	ab193349	1:1000	Goat Anti-mouse IgG (H+L)	HRP	Bio-Rad (Mississauga, Canada)	170-6516	1:2000	
**WB**	CD81	Antni-CD81 rabbit polyclonal	System Biosciences	EXOAB-CD81A-1	1:1000	Goat Anti-rabbit IgG (H+L)	HRP	Bio-Rad (Mississauga, Canada)	1706515	1:2000	
**WB**	GM130	Anti-GM130 rabbit polyclonal	Abcam (Toronto, Canada)	Ab52649	1:1000	Goat Anti-rabbit IgG (H+L)	HRP	Bio-Rad (Mississauga, Canada)	1706515	1:2000	
**WB**	P-gp	Anti-P-gp rabbit polyclonal	Abcam (Toronto, Canada)	ab129450	1:1000	Goat Anti-rabbit IgG (H+L)	HRP	Bio-Rad (Mississauga, Canada)	1706515	1:2000	
**WB**	CTTN	Anti-CTTN rabbit monoclonal	Abcam (Toronto, Canada)	ab81208	1:1000	Goat Anti-rabbit IgG (H+L)	HRP	Bio-Rad (Mississauga, Canada)	1706515	1:2000	
**WB**	RAB27A	Anti-RAB27A mouse monoclonal	Thermofisher (Canada)	89333209	1:1000	Goat Anti-mouse IgG (H+L)	HRP	Bio-Rad (Mississauga, Canada)	170-6516	1:2000	
**iEM**	pGSN	Anti-pGSN mouse monoclonal	Abgent (San Diego, US)	AM1936a	1:100	Goat-anti-mouse-IgG	Colloidal gold	Jackson (PA, USA)	115-215-068	1:50	
	Nuclei	Dapi									
	EVs	Exoquick Ultra EV isolation kit	System Biosciences	EQULTRA-20A-1							
**EM**		Glutaraldehyde	EMS (PA, USA)	16220							
**EM**		Paraformaldehyde	EMS (PA, USA)	15710							
**EM**		Sodium cacodylate trihydrate	EMS (PA, USA)	12300-25							

Table S4. Information on OVCA cell lines.

Cell line	Tumor origin	TP53 status	Other	Chemosensitivity
**A2780s**	Ovarian endometroid adenocarcinoma	Wild-type	PTEN/ARID1A	Sensitive
**A2780cp**	Ovarian endometroid adenocarcinoma	Mutant V127F, R260S	PTEN/ARID1A	Resistant
**OV90**	High grade serous ovarian cancer	Mutant Ser215Arg	None Detected	Resistant
**TOV3133G**	Serous-papillary adenocarcinoma	Nonsense	None Detected	Sensitive
**TOV3041G**	Serous adenocarcinoma	Wild-type	None Detected	Sensitive

Information on OVCA cell lines: The characterization of these cell lines have been verified in previous literature (Anglesio et al.,2013; Leroy et al., 2014; Provencher et al., 2000; Fleury et al., 2015; Letourneau et al., 2012; Ouellet et al., 2008;).

Table S5. Customized siRNA oligonucleotide duplexes.

Product	Target	Species	Company	Catalog #	Target sequence	Anti-sense sequence	Position on mRNA
siRNA 1	pGSN	Human	IDT (Iowa, USA)	N/A	GCGACCCGAGGCCGCGGCU	AGCCGCGGCCUCGGGUCGC	12
siRNA 2	pGSN	Human	IDT (Iowa, USA)	N/A	UGCCCGAGGCGCGGCCCAA	UUGGGCCGCGCCUCGGGCA	192
